# Posterior localization of ApVas1 positions the preformed germ plasm in the sexual oviparous pea aphid *Acyrthosiphon pisum*

**DOI:** 10.1186/2041-9139-5-18

**Published:** 2014-05-09

**Authors:** Gee-way Lin, Charles E Cook, Toru Miura, Chun-che Chang

**Affiliations:** 1 Laboratory for Genetics and Development, Department of Entomology/Institute of Biotechnology, College of Bioresources and Agriculture, National Taiwan University, No. 27, Lane 113, Roosevelt Road, Sec. 4, Taipei 106, Taiwan; 2 EMBL-European Bioinformatics Institute, Wellcome Trust Genome Campus, Hinxton, Cambridge CB10 1SD, UK; 3 Laboratory of Ecological Genetics, Graduate School of Environmental Science, Hokkaido University, N10 W5, Kita-ku, Sapporo, Hokkaido 060-0810, Japan; 4 Research Center for Developmental Biology and Regenerative Medicine, National Taiwan University, Taipei 100, Taiwan; 5 Genome and Systems Biology Degree Program, National Taiwan University, Taipei 106, Taiwan

**Keywords:** Aphid, Asymmetric localization, Developmental plasticity, Germ cells, Germline specification, Vasa

## Abstract

**Background:**

Germline specification in some animals is driven by the maternally inherited germ plasm during early embryogenesis (inheritance mode), whereas in others it is induced by signals from neighboring cells in mid or late development (induction mode). In the Metazoa, the induction mode appears as a more prevalent and ancestral condition; the inheritance mode is therefore derived. However, regarding germline specification in organisms with asexual and sexual reproduction it has not been clear whether both strategies are used, one for each reproductive phase, or if just one strategy is used for both phases. Previously we have demonstrated that specification of germ cells in the asexual viviparous pea aphid depends on a preformed germ plasm. In this study, we extended this work to investigate how germ cells were specified in the sexual oviparous embryos, aiming to understand whether or not developmental plasticity of germline specification exists in the pea aphid.

**Results:**

We employed *Apvas1*, a *Drosophila vasa* ortholog in the pea aphid, as a germline marker to examine whether germ plasm is preformed during oviparous development, as has already been seen in the viviparous embryos. During oogenesis, *Apvas1* mRNA and ApVas1 protein were both evenly distributed. After fertilization, uniform expression of *Apvas1* remained in the egg but posterior localization of ApVas1 occurred from the fifth nuclear cycle onward. Posterior co-localization of *Apvas1*/ApVas1 was first identified in the syncytial blastoderm undergoing cellularization, and later we could detect specific expression of *Apvas1*/ApVas1 in the morphologically identifiable germ cells of mature embryos. This suggests that *Apvas1*/ApVas1-positive cells are primordial germ cells and posterior localization of ApVas1 prior to cellularization positions the preformed germ plasm.

**Conclusions:**

We conclude that both asexual and sexual pea aphids rely on the preformed germ plasm to specify germ cells and that developmental plasticity of germline specification, unlike axis patterning, occurs in neither of the two aphid reproductive phases. Consequently, the maternal inheritance mode implicated by a preformed germ plasm in the oviparous pea aphid becomes a non-canonical case in the Hemimetabola, where so far the zygotic induction mode prevails in most other studied insects.

## Background

Studying model organisms can expand our knowledge in developmental biology and enrich our understanding of developmental diversity. One issue, developmental plasticity, has long been a difficult subject to access in animal models displaying little phenotypic plasticity. However, the pea aphid *Acyrthosiphon pisum*, a hemimetabolous hemipteran insect with abundant adaptive capacity in response to specific environmental cues, has proven an excellent model for study, and the recently published genome has made research on this species more accessible [1,2]. With a sequenced genome, scientists can start exploring - at molecular and systematic levels - how and why a genome can direct the alteration of phenotypic and reproductive traits by stimuli from outside. For example, environmental stresses such as high population density or predation trigger unwinged aphid females to produce winged offspring for dispersal [3,4], and aphid females also sense changing photoperiods to alter reproductive modes [5-7]. However, how the environmental signals are received by the genome and where the sequences required for polyphenic switches are located in the genome remain largely unknown.

Embryogenesis in aphid life cycles can be categorized as two types: asexual viviparous (bringing forth living young without fertilization) and sexual oviparous (producing fertilized eggs). In the remaining paragraphs of this article we abbreviate ‘asexual viviparous’ and ‘sexual oviparous’ to ‘viviparous’ and ‘oviparous’ , respectively. Recent studies suggest that there are two developmental programs underlying early development of the viviparous and oviparous embryos in the pea aphid. For example, the expressions of axis patterning genes (*hunchback*, *orthodenticle*, *caudal*, *nanos*) and the terminal gene *torso-like* display distinctly different patterns during early development in viviparous compared to oviparous embryos [8-11]. Comparison of transcriptomes of synchronized embryos in asexual and sexual phases shows that more than 30 genes are differentially transcribed, further supporting the existence of divergent programs of development underlying these two reproductive cycles [12]. It appears that the change of developmental programs occurs during the transition period from asexual to sexual phases - our previous work indicates that the germline-specific identity of *Api-piwi6* and *Api-ago3a*, both of which encode components of the Piwi-interacting RNA (piRNA) pathway, is lost from viviparity to oviparity [13]. Accordingly, we would like to know whether embryonic development is globally changed in distinct reproductive phases or whether some developmental mechanisms remain conserved.

Previously we identified posterior localization of Vasa (Vas) and Nanos (Nos), two of the most conserved germline markers in animals, in the syncytial blastoderm of the asexual aphid [14]. After cellularization, signals of Vas and Nos detected using cross-reacting antibodies were specifically recruited into the primordial germ cells (PGCs), suggesting that germline specification in the asexual aphid relies on a germ plasm preformed within the egg prior to cellularization [14]. However, we have not found any published descriptions of the assembly of maternal germ plasm in oocytes or early embryos in the sexual phase. To date, early segregation of germ cells in oviparous embryos has been reported in just two species: the black willow aphid *Melanoxanthus spp*. [15] and the spring grain aphid *Toxoptera graminum*[16]. In the black willow aphid, newly segregated ‘primitive germ cells’ were identified in the posterior region of the invaginating blastoderm. In the spring grain aphid, germline segregation was observed slightly later during gastrulation and segregated germ cells were located aside the posterior abdomen of the elongating germ band (embryo proper). In both aphid species, the presence of germ (polar) granules - an ultrastructural feature of the preformed germ plasm - was not described. Taken together, this suggests that germline specification in oviparous aphids is not germ-plasm dependent and, if this is the case, aphids may adopt two programs to specify germ cells in asexual and sexual phases.

In some existing animal models such as *Drosophila melanogaster* (fly), *Caenorhabditis elegans* (nematode), *Danio rerio* (zebrafish), and *Xenopus laevis* (frog), it is clear that specification of germ cells is driven by the maternal germ plasm assembled within the developing oocytes (reviewed in [17,18]). Removal of maternal germ plasm leads to the loss of PGCs or sterility in adults [19-21]. In *Mus musculus* (mouse), however, germline specification occurs much later during gastrulation and there is no maternally inherited germ plasm. Instead, specification of germ cells is triggered via induction of signals (the ‘induction mode’): Bmp4, Bmp8b, and Bmp2, members of the bone morphogenetic protein family, are secreted from the extra-embryonic ectoderm to induce certain cells in the neighboring proximal epiblast - the primary ectoderm - to form the precursors of PGCs [22-24]. After PGC precursors migrate to the posterior primitive streak, germline fate is further specified through the function of Blimp1, a key transcription factor known to repress the somatic program in the PGCs [25]. Although cytoplasmic inheritance of maternal germ plasm, the ‘inheritance mode’, is prevalent in most current animal models, the induction mode actually appears more common, and is likely ancestral, when other published data about germline development in emerging and non-model animals are collated (reviewed in [26]).

Among insect species, the inheritance mode for germline specification is mostly confined to Holometabola such as flies, mosquitoes, and wasps [27,28], all of which undergo complete metamorphosis. The lack of evidence to support the existence of maternal germ plasm in some holometabolous species like *Bombyx mori* (silkworm) [29,30], *Apis mellifera* (honeybee) [31,32], and *Tribolium castaneum* (beetle) [33,34], however, indicates that the inheritance mode is not a universal mechanism in Holometabola. By contrast, evidence from classical histological studies argues that insects adopting the induction mode are the majority in Hemimetabola, which in contrast to Holometabola undergo incomplete metamorphosis and are less derived. Ubiquitous expression of conserved germline genes such as *vas*, *piwi*, and *nos* in oocytes and early embryos of Hemimetabola further supports the above conclusion [35-37]. A recent report concerning the abolishment of PGC formation via knockdown of the mesodermal marker *twist* in the cricket *Gryllus bimaculatus* - a model for the basally branching insects - suggests the presence of signal induction for germline specification in hemimetabolous insects [35], though the actual signaling molecules are not yet identified.

Current data show that germline specification in Hemiptera is diverse. As described above, we have identified a presumptive germ plasm assembled in the posterior syncytial blastoderm of the viviparous pea aphid - a hallmark of the inheritance mode [14]. In the milkweed bug *Oncopeltus fasciatus* (Hemiptera: Lygaeidae), however, asymmetric localization of germline genes is not detected in oocytes and early embryos, and the segregation of PGCs is not identified until the late blastoderm stage. Moreover, knockdown of the conserved germline markers *vas* and *tudor* does not affect the formation of PGCs. All of this evidence implies the absence of a maternal germ plasm and the existence of an inductive mechanism in *Oncopeltus*[36]. Similar conclusions have also been described in germline development in other hemipteran species such as the assassin bug *Rhodnius prolixus* (Hemiptera: Reduviidae) [38] and several species of scale insects [39,40]. Accordingly, the germ plasm-dependent mechanism found in the viviparous pea aphid so far appears to be a non-canonical case in the Hemiptera in contrast to most other hemipterans. Recent studies show that the oviparous embryos adopt conserved mechanisms to pattern early embryogenesis while the viviparous embryos use a distinct and less conserved mechanism. Given the gross morphological similarities in oviparous aphid development with other hemipterans [38-41], it would be interesting to know if germline specification is also similar.

In order to explore how germ cells are specified during oviparous development in the pea aphid, we employed whole-mount *in situ* hybridization of *Apvas1* (previously known as *Apvas*[42], a *Drosophila vas* ortholog in the pea aphid), which we successfully used previously to label germline cell fate in viviparous embryos [42]. However, asymmetric localization of *Apvas1* mRNA, unlike that of the Vas signals, was not identified in the viviparous pea aphid. In order not to miss detecting Vas protein restricted to the potential germ plasm, we produced an antibody against ApVas1 for immunostaining. Through comparing germline specification in the viviparous and oviparous embryos, we aimed to identify similarities and differences between the development of germ cells in both of the reproductive cycles. Most importantly, this would reveal whether aphids adopt one or two versions of mechanisms for specifying germ cells in their life.

## Methods

### Pea aphid culture

The pea aphid strain ApL, formerly known as Sap05Ms2, was collected in Hokkaido, Japan and reared on broad beans (*Vicia faba*) in growth chambers under a long-day photoperiod (16 h light/8 h dark) at 20°C [43]. Induction of the sexual phase was carried out under a short-day photoperiod (8 h light/16 h dark) at 15°C. Details of induction were described in Ishikawa *et al*. [44,45]. Fertilized eggs for embryonic assays were maintained under short-day photoperiod at 15°C for 42 days. Embryonic staging for oviparous and viviparous embryogeneses followed Miura *et al*. [46].

### Production and purification of antibody against ApVas1

In order to avoid the conserved motifs of the DEAD (Glu-Asp-Ala-Glu)-box protein family, highly divergent sequences in the N-terminus of ApVas1 were selected and synthesized to induce the ApVas1 antibody (Additional file 1: Figure S1A). This ApVas1 antigen contains 451 amino acids (aa) in the N-terminal region (aa 4-454). A translation template of *Apvas1* was cloned using a forward primer (5'-GATCAGATCTGGGTGGTTGGGATGATGAATCTGG-3', encoding GGWDDESG) and a reverse primer (5'-AATTGAATTCCTATTCTCTCTCTGGTTGTTCACGATCAC-3', encoding GDREQPERE). Products of the polymerase chain reaction (PCR) were then subcloned into the pET32a(+) vector (Novagen, San Diego, CA, USA) to produce a His-tagged fusion protein in *Escherichia coli* C41(DE3) cells. Affinity purified His-tagged ApVas1 protein in homogenized polyacrylamide gel slices was used to immunize rabbits. For antibody purification, antisera were passed through a column packed with the cyanogen bromide (CNBr)-activated Sepharose 4B (GE Healthcare, Uppsala, Sweden) coupled with a polypeptide containing 186 aa in the N-terminus of ApVas1 (aa 4-189) (Additional file 1: Figure S1B). After non-specific bindings were washed off, the ApVas1 antibody was eluted with 0.1 M Glycine at pH 2.5 and immediately neutralized to pH 7.0 using 1 M Tris pH 8.0. The neutralized ApVas1 antibody was dialyzed against 1× phosphate buffered saline (PBS) and then stored at -80°C in 50% glycerol.

### Fixation and dissection of oviparous eggs

Aphid eggs were dechorionated in a 1:1 solution of methanol and heptane for 1 min with vigorous shaking. The heptane-methanol solution was removed without disturbing the dechorionated eggs after they had sunk to the bottom of the 1.5 mL Eppendorf tube. Eggs were then sequentially washed with mixture of methanol and 4% paraformaldehyde using ratios of 3:1, 1:1, and 1:3. After that, they were fixed in 4% paraformaldehyde for 20 min with mild shaking on a rotator. Fixed embryos collected before the end of 2 dAEL (days after egg laying) could be directly stained with riboprobe or antibody. From 3 dAEL onward, however, the serosal membrane that would turn black during staining had to be removed using a tungsten needle before staining. For embryos collected by the end of 4 dAEL we did not separate them from the yolk, but we remove yolk for those collected from 5 dAEL to increase the accessibility of riboprobe or antibody to the embryos.

### *In situ* hybridization, immunostaining, and imaging

Whole-mount *in situ* hybridization (WISH) on oviparous embryos was performed according to the protocol previously applied to the viviparous ovarioles [47]. Before application of the riboprobe, further fixation of dechorionated eggs or dissected embryos using 4% paraformaldehyde for 4 h at 25°C was required. In order to increase the probe specificity, we synthesized a Digoxigenin (DIG)-labeled antisense riboprobe complementary to the 149 nucleotides located in the 5’ untranslated region (UTR) of *Apvas1*, assuming that cross-hybridization would not occur to *Apvas2*-*4* mRNAs. *In situ* hybridization showed that the expression pattern detected using the 149-bp *Apvas1* antisense riboprobe differed from the expressions of *Apvas2*-*4* (Additional file 2: Figure S2). This indicates that the 149-bp probe is *Apvas1*-specific and we have used it for all detections of *Apvas1* expression in this study. Sequences of the forward and reverse primers for amplifying the 5’ UTR of *Apvas1* are 5'- TTTGGCGGTGATGATGGAGAAG-3' and 5'-AATCGAGTCTAGGTGGCAACG-3', respectively. WISH was carried out at 66°C for 36 h.

Immunostaining followed the protocol of Chang *et al.*[14], where viviparous ovarioles were stained using cross-reacting antibodies against Vas and Nos. For oviparous embryos, an additional fixation using 4% paraformaldehyde for 30 min at 25°C was required before application of the primary antibody. Antibodies and their dilution ratios were: (1) primary antibodies: ApVas1 antibody (1:20), ApVas2-4 antibodies (1:20), mouse anti-actin antibody (1:500) (Sigma, St Louis, MO, USA); and (2) secondary antibodies: anti-rabbit IgG conjugated with Cy5 (Invitrogen, Paisley, UK), Alexa Fluor 488 goat anti-mouse (Invitrogen), biotinylated goat anti-rabbit IgG (Vector labs, Burlingame, CA, USA) (all diluted 1:500). Nuclear staining of embryos older than 2 dAEL used 4',6-diamidino-2-phenylindole (DAPI) (2 μg/mL) (Sigma) at 37°C for 2 h, but those collected within 48 hours after egg laying (hAEL) were stained using propidium iodide (PI) (10 μg/mL) (Invitrogen) at 37°C for 2 h to avoid interference by autofluorescence from the yolk. F-actin staining on oviparous ovarioles was performed with Rhodamine Phalloidin (1:40) (Invitrogen); however, this was carried out using the anti-actin antibody (Sigma) in embryos prefixed with solutions containing methanol.

Bright-field images were photographed using a BX51 microscope (Olympus, Melville, NY, USA) connected with a DP50 CCD camera (Olympus); fluorescence images were taken by the laser-scanning microscope Zeiss LSM510 META (Carl Zeiss, Jena, Germany).

### Western blot

**Figure 1:**
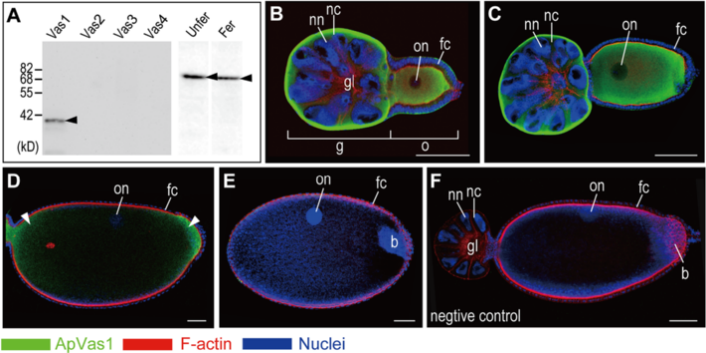
**Expression of ApVas1 in the oviparous ovarioles.** Each ovariole is composed of a germarium plus 1 to 2 egg chambers accommodating the developing oocytes. Color keys that indicate staining signals of ApVas1, F-actin (Rhodamine Phalloidin), and nuclear DNA (DAPI) in the ovariole are highlighted under the figure. Anterior of egg chamber is to the left. **(A)** Detection of ApVas1 *in vitro*: western blot. On a membrane blotted with the ApVas1-4 fusion proteins, only ApVas 1 was detected by the ApVas1 antibody. On another membrane blotted with the total protein extracted from unfertilized eggs and fertilized eggs, ApVas1 antibody detected a major band with the expected molecular weight (62.8 kD, arrowhead) of ApVas1. This major band could not have been ApVas2 (75.7 kD) and ApVas3 (71.7 kD), but it was close to ApVas4 (61.65 kD). **(B-E)** Detection of ApVas1 *in vivo*: immunostaining. **(B, C)** Previtellogenic oocytes. Oocyte in (C), with longer egg length, was more mature than that shown in (B). Expression of ApVas1 was evenly distributed in the cytoplasm of nurse cells (in germaria) and oocytes. Nuclei of nurse cells and oocytes were devoid of staining. **(D)** Vitellogenic oocytes. Preferential expression of ApVas1 (arrowheads) was identified in both anterior and posterior regions of the egg. **(E)** Mature oocytes subjected to oviposition. Signals of ApVas1 were almost not detected. Invading bacterial endosymbionts (bacteria) were observed in the egg posterior. **(F)** Negative control. Antibody against ApVas1, the primary antibody, was not applied to staining. Abbreviations: b, bacteria; fc, follicle cells; Fer, fertilized eggs; g, germaria; gl, germarial lumen; kD, kilodalton; nc, nurse cells; nn, nurse-cell nuclei; o, oocytes; on, oocyte nuclei; Unfer, unfertilized eggs. Scale bars: 100 μm.

About 50 ng of each ApVas fusion protein was loaded for protein electrophoresis (Figure 1A). Unfertilized eggs after oviposition were collected from females without mating; fertilized eggs were collected in parallel within 16 hAEL. Protein extracted from 10 unfertilized/fertilized eggs was dissolved in 100 μL of 2× sample buffer (57.7 mM Tris, 10% Glycine, 2% SDS, 10% glycerol, 12.5 mM EDTA, 0.02% bromophenol blue 0.02%, 1% beta-mercaptoethanol) and 6 μL of extracts was loaded onto each lane (Figure 1A). Primary antibody: anti-ApVas1 antibody (1:1,000); secondary antibody: goat peroxidase-labeled antibody against rabbit IgG (1:2,000) (Kirkegaard and Perry Laboratories (KPL), Gaithersburg, MD, USA). Signals were developed using the VisGlow™ Chemiluminescent Substrate, Horseradish Peroxidase (HRP) system (Visual Protein, Taipei, Taiwan).

## Results

### Maternal expression of *Apvas* genes in ovarioles of the sexual female

The pea aphid has four *vasa* homologs (*Apvas1*-*4*), all of which encode an ATP-dependent RNA helicase of the DEAD-box protein family [48]. For detecting the expressions of *Apvas* mRNA and protein, we synthesized antisense riboprobes of *Apvas1*-*4* and antibodies against ApVas1-4, respectively. Among the four *Apvas* genes, *Apvas1* is the sole germline marker throughout viviparous development while expressions of *Apvas2*-*4* are not germline specific (Additional file 2: Figure S2) [2,42]. We therefore examined the expression of *Apvas1* in the sexual phase, assuming that it was also a germline marker in the oviparous embryos.

*In situ* hybridization results showed that none of the four *Apvas* mRNAs, including *Apvas1*, was asymmetrically localized in the oocytes (Additional file 3: Figure S3). In order to examine whether ApVas1 was asymmetrically localized or uniformly distributed, we stained the ovarioles with the affinity-purified antibody against ApVas1. Purification of this antibody was specifically carried out using an immobilized antigen composed of 186 amino acids in the N-terminal region of ApVas1 to avoid antisera cross-reacting with downstream sequences that contain the eight conserved motifs of the DEAD-box protein family that is in all ApVas proteins (Additional file 1: Figure S1B). Western blots showed that the ApVas1 antibody could specifically detect ApVas1 and a major protein with expected size of the full-length ApVas1 (62.8 kD) from the unfertilized and fertilized eggs collected within 16 hAEL (Figure 1A). This suggests: (1) the affinity-purified antibody is specific to ApVas1; and (2) the newly laid eggs inherit maternal ApVas1 from the ovaries.

Immunostaining was performed on dissected ovarian tubules (ovarioles) encompassing the developing oocytes. In order to cover all developmental stages of oogenesis, we stained ovarioles dissected from both nymphs and adults. The nymphal aphids contain a higher proportion of previtellogenic oocytes whilst many more vitellogenic oocytes can be obtained from the adult aphids. Staining results showed that ApVas1 was uniformly expressed in the cytoplasm of nurse cells and that of the previtellogenic oocytes (Figure 1B, C). In the vitellogenic oocytes, we detected transient localization of ApVas1 in both anterior and posterior regions (Figure 1D). We conclude that localized signals observed in the vitellogenic oocytes were ApVas1: (1) localization of ApVas1 was not detected in oocytes that were not stained using the ApVas1 antibody (negative control, Figure 1F); and (2) expression patterns of ApVas2-4 (Additional file 4: Figure S4) were different from those of ApVas1 (Figure 1B-E). In mature oocytes subjected to oviposition, ApVas1 expression became undetectable (Figure 1E). We infer that the enlargement of egg chambers dilutes the intensity of the ApVas1 signal, because a major band corresponding to the size of ApVas1 could still be detected on the western blot (Figure 1A).

### Posterior localization of ApVas1/*Apvas1* in the syncytial and cellularized blastoderm

**Figure 2:**
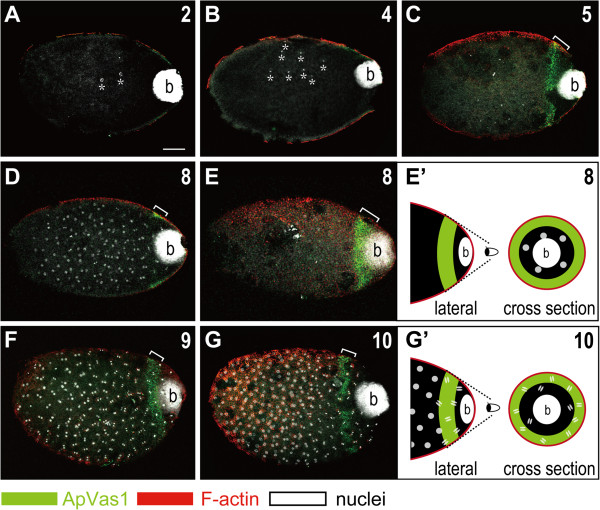
**Localization of ApVas1 in the posterior region of newly laid eggs.** Staining was performed on fertilized eggs collected within 16 hAEL. From 0 to 16 hAEL, embryos underwent 10 consecutive nuclear divisions. In each panel the nuclear cycle is highlighted in the upper right corner. DNA staining of bacteria (b) was detected in the posteriormost region of all egg chambers. **(A, B)** Second to fourth nuclear divisions. Asterisks mark the locations of cleaved nuclei in the shown focal plane. Localization of ApVas1 was not visualized. **(C)** Fifth nuclear division. Localization of ApVas1 (bracket) was first identified in the posterior region, anteriorly to the bacteria. **(D, E)** Eighth nuclear division. (D) and (E) are the same preparation but shown at two different focal planes. In (D), with the middle region of the egg in focus, most cleaved nuclei were observed but the ApVas1 stripe (bracket) was barely detected. In (E), with the egg cortex in focus, nuclear staining in the middle region was absent but the ApVas1 stripe (bracket) could be clearly identified. **(E’)** Schematic illustration indicating that cleaved nuclei have not reached the cortex region where ApVas1 is localized. **(F, G)** Ninth and tenth nuclear divisions. In contrast to eggs undergoing the eighth nuclear cycle, dividing nuclei could be observed in the cortex from the ninth nuclear cycle onward. In addition, the divisions began to progress in waves - nuclei located in the posterior third of the egg proceeded into anaphase but other nuclei remained at interphase or prophase. **(G’)** Schematic illustration indicating that some cleaved nuclei have reached the cortex region, being co-localized with the ApVas1 signals. Scale bars: 100 μm.

In the newly laid eggs, we did not identify localized signals of ApVas1 before the completion of the fourth cycle of nuclear division (Figure 2A, B). At the time that we first observed localized ApVas1 (fifth nuclear cycle, Figure 2C), energid nuclei had not yet reached the embryonic surface, suggesting that ApVas1 is not zygotically synthesized and is more likely maternally provided. By the end of the eighth cycle, we did not identify any energid nuclei on the cortex (Figure 2D, E). Migration of nuclei to the inner periphery of the egg - a hallmark of the formation of the syncytial blastoderm - was first identified from the ninth nuclear division onward (Figure 2F). Hence, we could find dividing nuclei within the ApVas1 stripe (Figure 2F, G). As previously described by Miura *et al.*[46], we also identified a mitotic wave along the anteroposterior axis from the ninth to tenth nuclear cycles, which displayed asynchronous nuclear divisions in different regions of the egg. However, localization of ApVas1 remained unaffected adjacent to the endosymbiotic bacteria (bacteria) at the posterior end of the egg, regardless of the status of nuclear divisions within the ApVas1 stripe (Figure 2F, G, G’).

**Figure 3:**
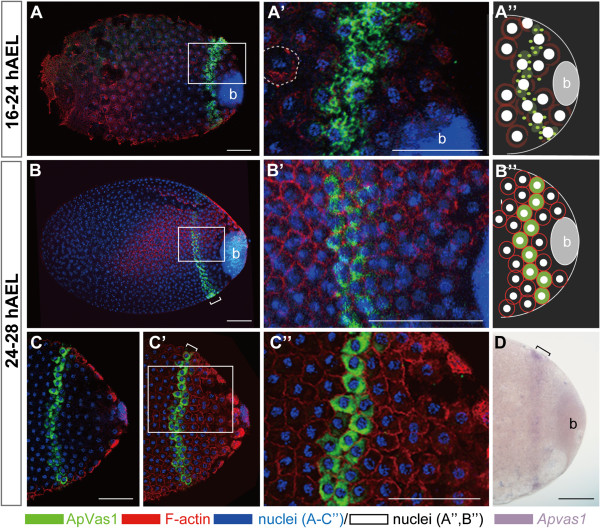
**Posterior localization of ApVas1 during cellularization of the syncytial blastoderm.** Staining with ApVas1 antibody was performed on eggs collected within 16 to 28 hAEL. Embryo presented in (D) was hybridized with the *Apvas1* antisense riboprobe. Status of cellularization was monitored by the expression of the polymerized F-actin using an anti-actin antibody. Anterior of egg chamber is to the left. Bacteria (b) are located in the posteriormost region of the eggs. **(A-A”)** Eggs collected during 16 to 24 hAEL. Actin caps (dashed circle) were visualized. In (A), localization of ApVas1 remained in the posterior cortex, forming a stripe anterior to the bacteria. (A’) is a magnification of the inset in (A): expression of ApVas1 was identified in the cytoplasm associated with the energid nuclei. (A”) Schematic illustration of ApVas1 expression shown in (A) and (A’). **(B-D)** Eggs collected during 24 to 28 hAEL. In (B), localization of ApVas1 remained in the egg posterior. (B’) is a magnification of the inset shown in (B): F-actin was located at the inner periphery of the forming cell membrane. (B”) Schematic illustration of ApVas1 expression shown in (B) and (B’). Embryos in (C) and (C’) belong to the same preparation but are shown at different focal planes. In (C), where the focal plane was internal, activity of F-actin was not detected; however, F-actin expression was detected in the cortex region shown in (C’). (C”) is a magnification of the inset shown in (C’): the intensity of ApVas1 and F-actin, in contrast to (B’), is increased. This suggests that the embryo in (C) is more mature than that in (B). (D) Posterior localization of *Apvas1* mRNA. Transcripts of *Apvas1* were restricted within a cortical stripe in the posterior region of the egg. Brackets in (C’) and (D) highlight locations of ApVas1/*Apvas1* enrichment. Scale bars: 100 μm.

In order to monitor the status of cellularization, we co-stained embryos using an antibody against the filamentous actin (F-actin). In insects such as *Drosophila*, crickets, and grasshoppers, formation of the ‘actin cap’ that surrounds each superficial energid nucleus has been regarded as a sign of cellularization and after cellularization F-actin is located to the inner periphery of the cell membrane [49-51]. We adopted these conserved features of F-actin polymerization to monitor cellularization in the pea aphid. During 16 to 24 hAEL, morphology of the actin caps was visualized and meanwhile we identified the expression of ApVas1 associated with the cytoplasm surrounding the energid nuclei (Figure 3A-A”). In the time window between 24 and 28 hAEL, we found that ApVas1 was incorporated by the forming cell membrane whose inner periphery was enriched with F-actin (Figure 3B, B’ , C’ , C”). We note that cellularization may not have been completed because within the interior region of the ApVas1-positive energids signals of F-actin staining are not detected (Figure 3C).

**Figure 4:**
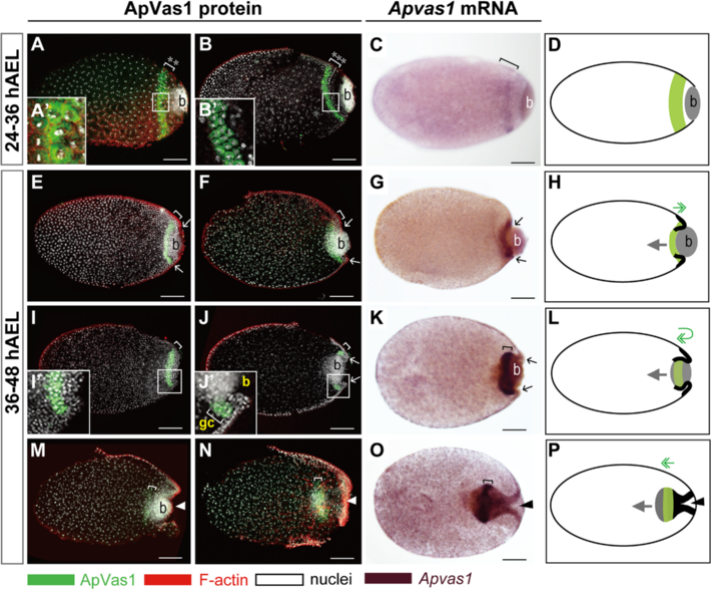
**Expression of ApVas1/*Apvas1 *during germ band formation.** (A, B, E, F, I, J, M, N) ApVas1 antibody staining; (C, G, K, O) *Apvas1 in situ* hybridization; ApVas1/*Apvas1* stripes: brackets; (D, H, L, P) Schematic illustrations: migration of bacteria and ApVas1/*Apvas1*-positive cells are indicated with grey arrows and green double arrows, respectively; invaginating blastoderm is marked with bold lines. Anterior of egg chamber is to the left; in (M-P), anterior of the germ band is to the right. **(A-D)** Blastoderm embryos prior to invagination. (A’) and (B’) are magnifications of insets in (A) and (B), respectively. Increasing number of ApVas1-positive cells was evidenced by the expansion of stripe width (A: 2 rows; B: 3 rows; cell rows: asterisks). (C) *Apvas1* mRNA was restricted to the same area as that of ApVas1 expression presented in (B). **(E-H)** Invagination of the posterior blastoderm. Invagination furrows marked with arrows in (F) and (G) appear more prominent than those in (E). Posterior boundary of the ApVas1/*Apvas1* stripe moved to encounter the anterior border of the bacteria. **(I-L)** Co-migration of bacteria and the ApVas1/*Apvas1* stripe into the yolk. Embryos in (I) and (J) belong to the same preparation but are shown at different focal planes: (I), cortex; (J), interior. (I’) and (J’) are magnifications of insets in (I) and (J), respectively. (K) *Apvas1* expression. (L) ApVas1/*Apvas1* stripe first reaches to the posteriormost end of the egg and then migrate back inside the yolk. After that, they follow the migration of bacteria. **(M-P)** Immersion of the ApVas1/*Apvas1* stripe into the yolk during anatrepsis. (M) Continuous invagination of the posterior blastoderm pushed bacteria and ApVas1 stripes toward the central region of the egg chamber. (O, N) Cells expressing ApVas1/*Apvas1* and bacteria were located at the posterior region of the germ band. Abbreviation: b, bacteria; gc, germ cells. Scale bars: 100 μm.

Apart from the ApVas1 protein, we also monitored the expression of *Apvas1* mRNA in parallel. *In situ* results showed that *Apvas1* transcripts were ubiquitously distributed in germaria, oocytes (Additional file 3: Figure S3A-C, F), and eggs collected within 24 hAEL (Additional file 5: Figure S5A). Posterior localization of *Apvas1* was not identified until 28 hAEL (Figure 3D). Like posterior localization of ApVas1 in embryos collected within the same developmental period (24 to 28 hAEL; Figure 3C, C’), *Apvas1* mRNA was enriched to a stripe area within the egg posterior (Figure 3D). Although we have not found optimal conditions to perform dual immunostaining and *in situ* hybridization on pea aphid embryos, the similar locations of the ApVas1 and *Apvas1* stripes (Figure 3C’ , D) and the synchronous migration of ApVas1/*Apvas1*-positive cells in later embryogenesis (Figure 4) suggest that ApVas1 and *Apvas1* are co-localized.

### Migration of cells expressing ApVas1/*Apvas1* during germ band formation

Prior to invagination of the blastoderm, we observed the increase of ApVas1-positive cells. Immunostaining results showed that the ApVas1 stripe expanded from two to three cells in width, and the total cell numbers increased from about 40 to 160 within the stripe (Figure 4A, A’ , B, B’). Initiation of invagination could be visualized by the appearance of invaginating furrows in the posterior region of the eggs (Figure 4E, F). According to our observations, invagination of the blastoderm formed the blastopore, pulled bacteria into the interior region of the egg, and meanwhile dragged the ApVas1 stripe on the cortex to migrate further posteriorly toward the posterior pole of the egg (Figure 4E, F, H). When bacteria became fully immersed within the yolk, the ApVas1 stripe located to the posterior cortex followed the movement of bacteria, migrating backward to the inner region of the egg (Figure 4I-L). Later, bacteria and ApVas1-positive cells further migrated toward the egg center (Figure 4N-P). Here, we confirm that PGCs travel over, rather than through, the bacteria. Taking embryos presented in Figure 4I and J as an example, these two embryos belong to the same preparation but are shown at different focal planes (4I, I’: cortex; 4 J, J’: interior region). If PGCs really traveled through the bacteria, we would not be able to observe an integrative stripe composed of ApVas1 positive cells without the interference of bacteria (Figure 4I) or the absence of ApVas1 positive cells within the bacteria (Figure 4J).

Like ApVas1, corresponding patterns of migration were also identified in the migration of *Apvas1*-positive cells during blastoderm invagination (Figure 4C, G, K, O). The chromogenic *in situ* results, moreover, allowed us to visualize the morphology of the invaginating blastoderm more clearly (Figure 4O), because under a bright-field microscope the shape of the blastoderm was not masked by the adjacent bacterial mass that emitted strong nuclear signals under a confocal microscope (Figure 4M, N). Identical patterns of blastoderm invagination and germ band formation have been described in embryogenesis of the black willow aphid *Melanoxanthus spp*. [15] and the spring grain aphid *T. graminum* (Additional file 6: Figure S6C) [16], suggesting that movement of Vas/*vas*-positive cells is likely conserved among the aphid species.

### Migration of cells expressing ApVas1/*Apvas1* during germ band extension, katatrepsis, and late embryogenesis

**Figure 5:**
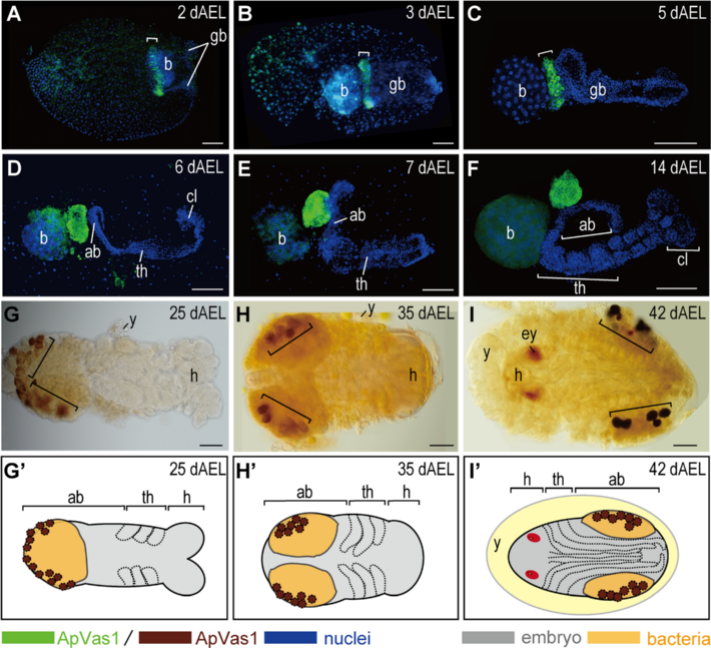
**Expression of ApVas1 during germ band extension, katatrepsis, and late embryogenesis.** Dissected embryos were collected from 2 to 42 dAEL. Before katatrepsis (A-H): embryonic anterior, right; after katatrepsis (I): embryonic anterior, left. ApVas1 stripes: brackets. **(A-C)** Germ band extension. (A) Embryos at 2 dAEL of development. ApVas1 stripe and bacteria were co-localized to the posteriormost region of the germ band. (B, C) Embryos at 3 to 5 dAEL of development. Bacteria migrated through the ApVas1 stripe, locating posteriorly to the ApVas1 stripe. **(D-F)** Morphogenesis of the germ band. (D, E) Embryos at 6 to 7 dAEL of development. Structures of the cephalic lobe, thorax, and abdomen could be identified. Cephalic lobe in (E) was truncated; see Additional file 8: Figure S8B, D for intact morphology. (F) Embryos at 14 dAEL of development. Segments in the head and thorax were visible. During 6 to 14 dAEL, the ApVas1 stripe was reorganized into a globular shape. **(G-I)** Fully-segmented embryos before and after katatrepsis. Dorsal view of embryos attached with bacteria is presented, and follow the style of Miura *et al*. and Shingleton *et al*. [46,52]. (G) Embryos at 25 dAEL of development. ApVas1-positive cells associated with the bacteria were located to the posteriormost region of the embryo, forming a ‘U’ shaped cap. (H) Embryos at 35 dAEL of development. ApVas1-positive cells were bilaterally located in the abdomen. Another embryo at similar age in lateral view was shown in Additional file 7: Figure S7A. (I) Embryos at 42 dAEL of development (after katatrepsis). The distribution of ApVas1-positive cells remained similar to that seen in embryos of 35 dAEL. **(G’-I’)** Schematic illustrations shown in (G-I). Abbreviations: ab, abdomen; b, bacteria; cl, cephalic lobe; ey, eyes; gb, germ band; h, head; th, thorax; y, yolk. Scale bars: 100 μm.

Although bacterial cells were migrating ahead of the ApVas1/*Apvas1*-positive cells during germ band formation (Figure 4E-P), these two populations of cells reached the posterior end of the germ band that was about to elongate almost at the same time (Figure 5A). However, their co-localization broke up while the germ band continued to extend. The ApVas1-positive cells, as a consequence, became located between the bacteria and the elongating germ band (Figure 5B, C). We infer that further migration of bacteria and ApVas1-positive cells toward the egg anterior was driven by the extension of the germ band.

During germ band extension, the morphology of the cephalic lobe, thorax, and abdomen became distinct. Meanwhile, we found that the morphology of the ApVas1 stripe was also changed: the stripe was transformed into a globular structure, extra-embryonically located adjacent to the elongating abdomen (Figure 5D-F). When embryos were fully segmented and the limb buds became visible ApVas1-positive cells aggregated to the posterior region, forming a U shaped ring (Figure 5G, G’). Prior to katatrepsis (embryo flip), we found that ApVas1-positive cells, together with the bacteria, were separated into clusters within the dorsal side of the embryo (Figure 5H, H’; Additional file 7: Figure S7A, B). After katatrepsis, the ApVas1-positive cells were located bilaterally in the dorsal region of the abdomen (Figure 5I, I’) [46,52]. In parallel, we also examined the expression of *Apvas1* in the elongating germ band (Additional file 8: Figure S8A, B), finding that it was restricted to the same places where ApVas1 was expressed (Additional file 8: Figure S8C, D). This implies that in later stages of development *Apvas1* mRNA and ApVas1 protein are co-localized in the germ cells.

## Discussion

### *Apvas1* gene as a germline marker in both sexual and asexual females

The ultimate goal of this study is to understand how germ cells are specified in the oviparous pea aphid. In particular, we are interested in whether or not germline specification relies on a preformed germ plasm. Here, we employed *vas* as a germline marker to monitor the assembly of germ plasm and migration of germ cells. Although *vas* has been regarded as the most conserved germ-cell specific gene in animals, in some species *vas* mRNA and Vas protein are not concurrently localized to the preformed germ plasm. For example, in *Drosophila* it is Vas protein, rather than *vas* mRNA, that is localized to the germ (pole) plasm [53,54]. By contrast, in zebrafish preformed germ plasm within the cleavage planes is enriched in *vas* mRNA, rather than the uniformly expressed Vas protein, in two cell-stage embryos [55-58]. In order to maximize the opportunities for identifying the germ plasm in the oviparous pea aphid, we accordingly explored the expression of both *Apvas1* (mRNA) and ApVas1 (protein).

Among previous studies of embryogenesis in sexual females, germline development has been best characterized in *T. graminum* (spring grain aphid). According to Webster and Phillips [16], segregation of the ‘primitive germ cells’ - an old term for the primordial germ cells (PGCs) - in *T. graminum* was first detected extra-embryonically adjacent to the posterior abdomen of the elongated germ band. This location corresponds to where *Apvas1*/ApVas1 was expressed in embryos at equivalent developmental stage in the pea aphid (Figure 5D, E; Additional file 8: Figure S8). Specific expression of *Apvas1*/ApVas1, moreover, could be identified in germ cells that were morphologically identifiable in the dorsal region of embryos after katatrepsis (*Apvas1*: data not shown; ApVas1: Figure 5I, Additional file 7: Figure S7A). The above evidence suggests that *Apvas1*/ApVas1-positive cells are PGCs in the oviparous embryo. Taken together with our previous work showing that *Apvas1*[42] and ApVas1 [14] are also germline-specific in the viviparous embryo, we conclude that the *Apvas1* gene is a germline marker of pea aphids in both sexual and asexual phases.

### Identification and assembly of the preformed germ plasm in the sexual female

The preformed germ plasm is usually assembled in a subcellular area within the egg prior to cellularization. Therefore, asymmetric localization of germline markers that will be inherited by the PGCs has become a geographical signature of the preformed germ plasm (reviewed in [59]). In the oviparous embryo, we could detect the asymmetric localization of ApVas1 in the eggs prior to cellularization (Figure 2C-G) and monitor the incorporation of ApVas1 into presumptive PGCs during cellularization (Figure 3B’). After katatrepsis, locations of the ApVas1-positive PGCs (Figure 5I, I’) in the dorsal region of the embryos correspond to the morphologically identifiable germ cells in the viviparous aphids at equivalent developmental stages [42,46]. Accordingly, we conclude that the subcellular cytoplasm restricting ApVas1 expression in the posterior cortex of the early syncytial blastoderm is the preformed germ plasm (Figure 2C).

In early embryos of *Drosophila* and the pea aphid, Vas protein is localized to the germ plasm whereas *vas* mRNA is uniformly distributed (Figure 2C-G, Additional file 5: Figure S5A) [14,42,53,54]. This suggests that Vas protein, rather than *vas* mRNA, is involved in the assembly of germ plasm in both species. However, the timings of Vas localization are different: in *Drosophila* posterior localization of Vas to the germ plasm occurs during mid oogenesis, but in the oviparous embryos a subcellular cytoplasm containing ApVas1 expression - the presumptive germ plasm - cannot be identified through oogenesis (Figure 1B, C). Although in some vitellogenic oocytes posterior localization of ApVas1 is identified (Figure 1D), we do not consider this the onset of germ-plasm assembly because later ApVas1 localization cannot be continuously detected in the posterior region of mature oocytes subjected to oviposition (Figure 1E) and newly laid eggs (Figure 2A, B). Besides, locations of ApVas1 localization in the oocytes and embryos do not exactly correspond to each other - ApVas1 is localized to the posteriormost end of the oocyte (Figure 1D) while in the embryo the ‘ApVas1 stripe’ is already located anteriorly to the bacteria (Figure 2C).

Compared with *Drosophila*, the oviparous pea aphid segregates germ plasm much later: not until the fifth nuclear division after fertilization (Figure 2C). In other insects that adopt the inheritance mode, the assembly of germ plasm also takes place during oogenesis [27,28,60]. Thus the ‘post-fertilization’ assembly of germ plasm in the oviparous aphid is so far unusual. Nonetheless, a similar situation can be found in the nematode *C. elegans*, where fertilization triggers the posterior localization of maternal P granules (germ granules) [61]. At present we do not have experimental data to show whether the assembly of germ plasm in the oviparous aphid is fertilization dependent or independent. However, the segregation of germ plasm in the viviparous aphid does not depend on sperm entry [14]. If aphids of both reproductive phases share the same mechanism of germline specification then fertilization may not play a role in the assembly of germ plasm in the oviparous embryo. On the other hand, unless we can examine the distribution of all transcripts specific to germ cells, we cannot exclude the possibility that the germ plasm has already been assembled during oogenesis via depositing germline determinants to localize ApVas1, or locally translate *Apvas1*, after fertilization.

### Comparison of germline specification and migration in oviparous and viviparous pea aphids

Although distinct developmental programs have been identified in oviparous and viviparous embryogenesis [8,10,11], our data show that the germ-plasm dependent specification of germ cells is conserved in both modes of aphid development. The early segregation of germ plasm, we infer, allows germ cells to locate in an extra-embryonic position so that the germline fate can be isolated from the somatic body plans. At a molecular level, differential expression of duplicated germline genes (for example, *vas*, *nos*, *piwi*, and *ago3*) during the switch of reproductive cycles and the establishment of sexual/asexual-specific germline transcriptomes may be required for germline reprogramming [13], but not for the specification of germ cells. Consequently, germline specification - at the morphological level - does not display developmental plasticity in the pea aphid and perhaps in other aphid species as well.

**Figure 6:**
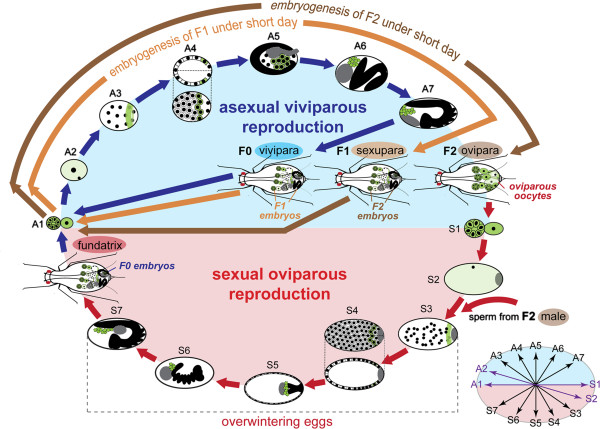
**Comparison of germline development during sexual and asexual reproductive phases.** Induction of reproductive modes relies on the length of photoperiods: long days induce the asexual phase; short days induce the sexual phase (for details see Methods). From asexual to sexual reproduction (F0 to F2), embryos pregnant by the F0 females and F1 females must develop under short light period days. Expression of ApVas1 in germaria, oocytes, presumptive germ plasm, and germ cells is designated in green. **(S1-S7)**: germline development during oviparous reproduction; **(A1-A7)**: germline development during viviparous reproduction. S1/A1, germaria and oocytes; S2/A2, mature oocytes; S3/A3, syncytial blastoderm; S4/A4, cellularized blastoderm (blastula); S5/A5, germ band formation; S6/A6, extending germ band; S7/A7, embryos undergoing katatrepsis. Corresponding stages of development are highlighted in the bottom right corner. Presumptive germ plasm is specified in the posterior syncytial blastoderm (S3/A3); cellularization of germ cells occurs in the posterior blastula (S4/A4); germ cells are migrating during mid embryogenesis (S5-S7/A5-A7). Migrating germ cells are settled in the putative gonads of late embryos in both reproductive phases. In the sexual phase, the oviparous embryo will give rise to the fundatrix (stem mother hatched from overwintering eggs). The asexual phase begins from the birth of fundatrices bearing viviparous embryos. Data resources of germline development: oviparous development, Figures 1, 2, 3, 4, and 5 and Additional file 3: Figure S3, Additional file 5: Figure S5, Additional file 7: Figure S7, Additional file 8: Figure S8; viviparous development: [14,42] and Additional file 2: Figure S2.

Additionally, we find that segregation of germ plasm and migration of germ cells in both oviparous and viviparous embryos follows a very similar pattern (Figure 6) [14,42]: (1) the preformed germ plasm expressing Vas protein is not identified during oogenesis (S1/A1; S2/A2); (2) segregation of the preformed germ plasm occurs in the posterior region of syncytial blastoderm (S3/A3); (3) PGCs form in the posterior blastoderm after cellularization (S4/A4); (4) after germ band formation, PGCs are located posteriorly to the germ band regardless of its orientation in the egg chamber (S5/A5); (5) during germ band elongation, PGCs are aggregated and located extra-embryonically aside the elongating abdomen (S6/A6); and (6) from katatrepsis onward, PGCs migrate toward the anteriormost region of egg chambers and afterwards they migrate posteriorly (S7/A7). However, we find that migrating PGCs are already grouped into clusters prior to katatrepsis in the oviparous embryos (Figure 5G, H) but in the viviparous embryos PGCs remain unseparated at the equivalent stage of development [42].

### Evolution and development of germline specification in aphids and other hemimetabolous insects

**Figure 7:**
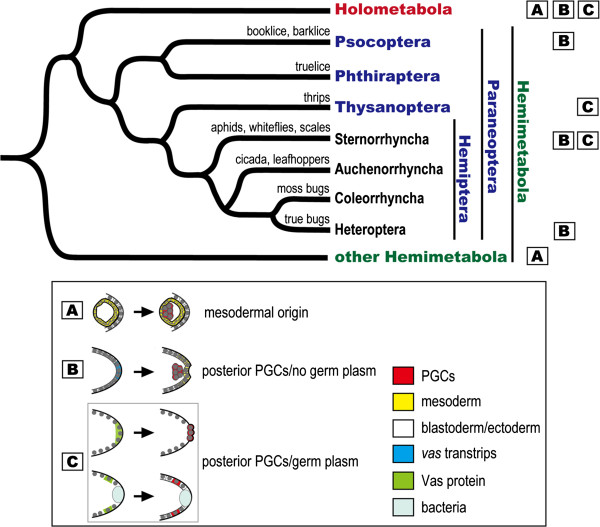
**Phylogenetic distribution of mechanisms for germline specification within the Paraneoptera.** In the phylogenetic tree we display four orders of the superorder Paraneoptera: Psocoptera (booklice, barklice), Phthiraptera (trulice), Thysanoptera (thrips), and Hemiptera (true bugs). For discussing germline specification within the Hemiptera, we present four suborders including Sternorrhyncha (aphids, whiteflies, scales), Auchenorrhyncha (cicadas, leafhoppers), Coleorrhyncha (moss bugs), and Heteroptera (true bugs). Squares A, B, and C designate mechanisms of germline specification: A, germ cells are derived from the mesoderm during mid/late embryogenesis; B, germ cells are segregated adjacent to the posterior blastoderm, which takes place after formation of the blastoderm, but a preformed germ plasm has not been identified; C, germline specification is driven by a preformed germ plasm located in the posterior pole (*Drosophila*) or slightly anterior to the posterior end (aphid) of the egg. With the exceptions of booklice (*Liposcelis divergens*; B) [62], thrips (*Haplothrips verbasci*; C) [63], and scale insects (*Pseudococcus mcdanieli*, *Lecanodiaspis pruinosa*, and *Icerya purchase*; B) [39], where study of germline segregation was carried out with traditional microscopic approaches, mechanisms of germline specification are deduced from expression data or functional analysis of the germline marker *vas*/Vas. Sources for molecular data: Hemiptera: aphids (*Acyrthosiphon pisum*; C) [14], true bugs (*Oncopeltus fasciatus*; B) [36,64]; other Hemimetabola: crickets (*Gryllus bimaculatus*; A) [35]; Holometabola: honeybees (*Apis mellifera*; A) [32], the flour beetle (*Tribolium castaneum*; B) [34], fruit flies (*Drosophila melanogaster*; C) [53]. Phylogenetic relationships of the Paraneoptera are based upon Grimaldi and Engel [65]; monophyletic clades within the Hemiptera are from Wheeler *et al.*[66] and Cryan and Urban [67]. Abbreviation: PGCs, primordial germ cells.

The preformed germ plasm, a morphological feature of the inheritance mode, has not been reported in any hemipterans apart from the aphids. For example, *Pseudococcus mcdanieli* (mealybug), *Lecanodiaspis pruinosa* (false pit scale), and *Icerya purchasi* (cottony cushion scale) are scale insects that all belong to the same suborder Sternorrhyncha as the aphids; however, in these species germ plasm was undetectable using classical histological approaches and segregated germ cells were first identified adjacent to the posterior blastoderm (Figure 7) [39]. In *Oncopeltus fasciatus* (milkweed bug), a true bug belonging to the suborder Heteroptera, how Vasa protein is distributed during early development has not been reported; nevertheless, mRNAs of 19 homologues to *Drosophila* germline genes are not asymmetrically localized during early development, and formation of germ cells is not affected by the knockdown of conserved germline markers [36].

Taken together, the above cases suggest that absence of the preformed germ plasm is the norm in the Hemiptera although how germ cells are specified in the other two hemipteran suborders Auchenorrhyncha and Coleorrhyncha remains unclear (Figure 7). Accordingly, the common ancestor of hemipterans likely employed the induction mode to specify germ cells and the presence of germ plasm in aphids might be the result of independent evolution within the Hemiptera. Nonetheless, we cannot exclude the possibility that the gain of a germ plasm in aphids occurred after the parthenogenetic phase was added to the sexual life cycle more than 200 million years ago (reviewed in [68]), but how the inheritance mode was adopted in the sexual phase is still an unanswered question.

Though the inheritance mode is common neither in Hemiptera nor in other orders of the Hemimetabola, this mechanism has also been reported in the mullein thrips *Haplothrips verbasci*, a member of the order Thysanoptera [63]. According to Heming, a putative germ (pole) plasm could be identified in the posterior end of the unfertilized egg and polar granules in the germ plasm can be inherited by the newly segregated germ cells after fertilization in *H. verbasci*[63]. Taking together with the germ plasm identified in the pea aphid, we find that both cases - one in the Hemiptera (aphid) and one in the Thysanoptera (thrips) - occur within the Paraneoptera, a monophyletic superorder of insects and a sister group to the Holometabola (Figure 7) (reviewed in [65]). This implies that the inheritance mode can be identified in derived orders of hemimetabolous insects and the gain of a preformed germ plasm is achieved via independent evolution.

## Conclusions

Identification of the preformed germ plasm in both oviparous and viviparous embryos of the pea aphid suggests that developmental plasticity of the germline-specification mode does not occur in the alternate reproductive cycles in the aphids. This differs from the distinct developmental programs known to regulate axis patterning in the asexual and sexual phases. The preformed germ plasm in the pea aphid so far is the first maternal germ plasm identified among hemimetabolous insects using molecular approaches, and thus provides an opportunity for studying how a germ plasm is assembled in the Hemimetabola. It has been clear that a homolog of *oskar* (*osk*), a molecular anchor known to localize Vas protein in the germ plasm of *Drosophila*, does not exist in the pea aphid genome [1]. Finding molecular anchors for ApVas1 localization in the germ plasm will thus enable us to understand how an ‘*osk*-independent’ molecular networking for germ plasm assembly functions in less derived insects.

## Abbreviations

Aa: amino acids; ab: abdomen; b: bacteria; cl: cephalic lobe; CNBr: cyanogen bromide; dAEL: day after egg laying; DAPI: 4',6-diamidino-2-phenylindole; DIC: differential interference contrast; DIG: digoxigenin; ey: eyes; fc: follicle cells; Fer: fertilized eggs; g: germaria; gb: germ band; gl: germarial lumen; h: head; hAEL: hour after egg laying; HRP: Horseradish Peroxidase; kD: kilodalton; nc: nurse cells; nn: nurse-cell nuclei; Nos: Nanos; o: oocyte; on: oocyte nuclei; osk: *oskar*; PBS: phosphate buffered saline; PCR: polymerase chain reaction; PGCs: primordial germ cells; PI: propidium iodide; piRNA: Piwi-interacting RNA; th: thorax; Unfer: unfertilized eggs; UTR: untranslated region; Vas: Vasa; WISH: whole-mount *in situ* hybridization; y: yolk.

## Competing interests

The authors declare that they have no competing interests.

## Authors’ contributions

GWL: collection and analysis of data, experimental design, manuscript writing, critical revision of the manuscript. CEC: data analysis, manuscript writing, and critical revision of the manuscript. TM and CC: conception and design, data analysis, training and financial support, critical revision of the manuscript. All authors read and approved the final manuscript.

## Supplementary Material

Additional file 1: Figure S1 Sequence alignment of the ApVas1-4 proteins. (A) Alignment of the N-terminal sequences of ApVas1-4. The aligned region includes divergent sequences as well as conserved amino acids in the helicase domain (green bar). Black boxes indicate amino acids identical to that of ApVas1; gray boxes indicate more than two amino acids that are identical between ApVas2-4 proteins. (B) Schematic comparison of ApVas1-4. Green boxes highlight the conserved helicase domain and blue bars mark the locations of the antigen sequences. The red bar beneath ApVas1 highlights the sequence region of the ligand that was used for affinity purifying the ApVas1 antibody. Click here for file

Additional file 2: Figure S2 Expression of *Apvas1*-*4* mRNA during viviparous development. Germaria, oocytes, and embryos in the ovariole were hybridized with the antisense riboprobes of *Apvas1*-*4*. Locations of germ cells are indicated with hollow arrowheads. Anterior of egg chambers is to the left. (A-E) *Apvas1*. (A) In germaria and oocytes, *Apvas1* mRNA was expressed in the cytoplasm of nurse cells and oocytes. (B) During nuclear divisions, expression of *Apvas1* remained in the cytoplasm of the syncytial blastoderm. (C-E) Expression of *Apvas1* was specifically restricted to the germ cells from blastoderm formation (stage 6) till late embryogenesis. (F-J) *Apvas2*. (F, G) Expression of *Apvas2* was undetectable in germaria, oocytes, and syncytial blastoderm, but in the follicle cells weak expression could be identified. (H, I) Except in the germ cells, transcripts of *Apvas2* were evenly distributed in embryos before katatrepsis. (J) In embryos after katatrepsis, expression of *Apvas2* was ubiquitous including the germ-cell region. (K-O) *Apvas3*. (K, L) Expression of *Apvas3* was not detected in germaria, oocytes, and syncytia (including the follicle cells). (M, N) Like *Apvas2* (panels H and I), uniform expression of *Apvas3* was detected except in the germ cells. (O) Expression patterns of *Apvas3* in late embryos are very similar to those of *Apvas2* (panel J). (P-T) *Apvas4*. (P, Q) Expression of *Apvas4* was detected in the germarial lumen, oocytes, and syncytial blastoderm. (R-T) *Apvas4* was uniformly distributed in germ cells and somatic cells after blastoderm formation onward. Abbreviations: ab, abdomen; b, bacteria; cl, cephalic lobe; fc, follicle cells; g, germaria; h, head; nc, nurse cells; o, oocytes; on, oocyte nuclei; th, thorax. Scale bars: 20 μm in (A-C, F-H, K-M, P-R), 50 μm in (D, I, N, S) and 100 μm in (E, J, O, T). Click here for file

Additional file 3: Figure S3 Expression of *Apvas1*-*4* mRNA in the oviparous ovarioles. (A-F) *Apvas1*. (A, B, C, F): antisense riboprobes; (D, E): sense riboprobes as negative controls. In germaria and previtellogenic oocytes, *Apvas1* mRNA was expressed in the cytoplasm of nurse cells and oocytes (A). During vitellogenesis, expression of *Apvas1* remained in the cytoplasm but the intensity of signals decreased as the egg chambers enlarged (B, C, F). (G-I) *Apvas2*. Expression of *Apvas2* was detected in germaria (G), previtellogenic oocytes (G), and early vitellogenic oocytes (H). In late vitellogenic oocytes (I), *Apvas2* mRNA was preferentially expressed in the cortex of oocytes as well as the nuclei. (J-L) *Apvas3*. Expression of *Apvas3* was detected in germaria (J), previtellogenic oocytes (J), and early vitellogenic oocytes (K). However, it was almost undetected in late vitellogenic oocytes (L). (M-O) *Apvas4*. Expression of *Apvas4* was detected in germaria (M), previtellogenic oocytes (M), early vitellogenic oocytes (N), and late vitellogenic oocytes (O). Anterior of egg chambers is to the left. Abbreviations: b, bacteria; -Ctrl, negative control; fc, follicle cells; g, germaria; gl, germarial lumen; nc, nurse cells; nn, nurse-cell nuclei; o, oocytes; on, oocyte nuclei. Scale bars: 100 μm. Click here for file

Additional file 4: Figure S4 Expression of ApVas2-4 proteins in the ovarioles dissected from the oviparous females. (A-C) ApVas2. (A) Signals of ApVas2 were not detected in the germaria and developing oocytes. (B, C) During vitellogenesis, expression of ApVas2 was detected in the oocytes and follicle cells. Development of signals was performed using the chromogenic substrate 3,3’-Diaminobenzidine (DAB) Liquid Substrate System (Sigma). (D-F) ApVas3. Expression of ApVas3 was detected in the nuclei throughout oogenesis. (D) Signals of ApVas3 were barely detected in the germaria. (E) ApVas3 expression was restricted to the follicle cells during mid vitellogenesis. (F) In the late vitellogenic oocytes, expression of ApVas3 was almost undetectable. (G-I) ApVas4. (G) Expression of ApVas4 was identified in germaria and previtellogenic oocytes. (H) ApVas4 was preferentially expressed in the oocyte posterior but, unlike ApVas3, ApVas4 expression was not detected in follicle cells and oocyte nuclei. (I) Signals of ApVas4 were ubiquitously distributed within the egg chamber but preferential expression of ApVas4 in the posterior region of oocytes was not detected. Color keys for staining signals of ApVas2-4 (immunostaining), F-actin (Rhodamine Phalloidin), and nuclear DNA (DAPI) are highlighted under the figure. Anterior of egg chambers is to the left. Abbreviations: b, bacteria; fc, follicle cells; g, germaria; nn, nurse-cell nuclei; o, oocytes; on, oocyte nuclei. Scale bars: 100 μm. Click here for file

Additional file 5: Figure S5 Expression of *Apvas1* mRNA in early oviparous embryos and late viviparous embryos. Embryos were hybridized with antisense riboprobes of *Apvas1*. (A) Oviparous eggs collected within 24 hAEL. *Apvas1* was ubiquitously expressed within the embryos. (B) Viviparous embryos at stage 18 of development. *Apvas1* marked germ cells settled in the dorsal region of embryos [42], showing that *Apvas1* is germline specific. This staining served as a positive control for probe quality and *in situ* conditions. Anterior of oviparous egg chambers and viviparous embryos are to the left. Abbreviations: b, bacteria; gc, germ cells; h, head. Scale bars: 100 μm. Click here for file

Additional file 6: Figure S6 Schematic illustrations of germ band formation in sexual females. Images in this figure are modifications of Webster and Phillips [16] and Tannreuther [15], where embryogenesis of the spring grain aphid *T. graminum* and the black willow aphid *Melanoxanthus spp*. (a synonym of *Pterocomma spp.*), respectively, are described. (A-C) Invagination of the blastoderm. (A) Before invagination, the single layer of blastodermal cells has been invaded by the endosymbiotic bacteria *Buchnera* from the posterior pole of the egg. (B) Invagination of the blastodermal cells occurs in the area flanking the bacteria. (C) Invaginating blastoderm and invading bacteria are both migrating into the egg chamber. (D-F) Formation of the germ band. (D) Invaginating blastoderm becomes thickened and extends further inside the yolk. Blastodermal cells that link to the bilateral invaginating blastoderm differentiate into serosal cells. (E, F) Both ends of the extending blastoderm fuse together, detaching from the serosal membrane. One side of the germ band further thickens to become the germ band; another side differentiates into the amnion. Location of ‘primitive germ cells (red)’ corresponds to that of the primordial germ cells expressing ApVas1/*Apvas1* (Figure 4M-O; Figure 5A-C). Abbreviation: am, amnion; b, bacteria; bl, blastoderm; e, energids; em, egg membrane; gb, germ band; ibl, invaginating blastoderm; PGCs, primordial germ cells; s, serosa; y, yolk. Click here for file

Additional file 7: Figure S7 Expression of ApVas1 in the oviparous embryos undergoing katatrepsis. Anterior of egg chamber is to the left; dorsal side of the embryo is lower. ApVas1-positive cells: brackets. (A) Lateral view of an embryo collected by the end of 35 dAEL. ApVas1-positive cells were bilaterally located in the dorsal region of the abdomen. (B) Magnification of ApVas1-positive cells from another embryo that was also collected by the end of 35 dAEL. As shown in the figure, there are 12 to 13 PGCs within each cluster (brackets). Abbreviations: h, head; th, thorax. Scale bars: 100 μm. Click here for file

Additional file 8: Figure S8 Expression of *Apvas1* mRNA and ApVas1 protein in oviparous eggs collected during 6 to 7 dAEL. From 6 dAEL onward, an enlarged cephalic lobe of the embryo was observed. In the images shown, cephalic lobes of embryos are to the right (though anterior of egg chambers is still to the left). (A, B) Embryos hybridized with antisense riboprobes of *Apvas1*. (A) Embryos at 6 dAEL. *Apvas1*-positive cells were aligned within a stripe between bacteria and the embryo (germ band). (B) Embryos at 7 dAEL. *Apvas1*-positive cells were aggregated as a ball-like shape. (C, D) Embryos were stained with antibody against ApVas1 protein. Expression patterns of ApVas1 (C, D) are very similar to those of *Apvas1* (A, B). Abbreviations: ab, abdomen; b, bacteria; cl, cephalic lobe; th, thorax. Scale bars: 100 μm. Click here for file
