# Supplementary figures and images for: Posterior localization of ApVas1 positions the preformed germ plasm in the sexual oviparous pea aphid Acyrthosiphon pisum
Source: EvoDevo. 2014 May 9;5:18. doi: 10.1186/2041-9139-5-18 (PMC4030528; doi:10.1186/2041-9139-5-18)

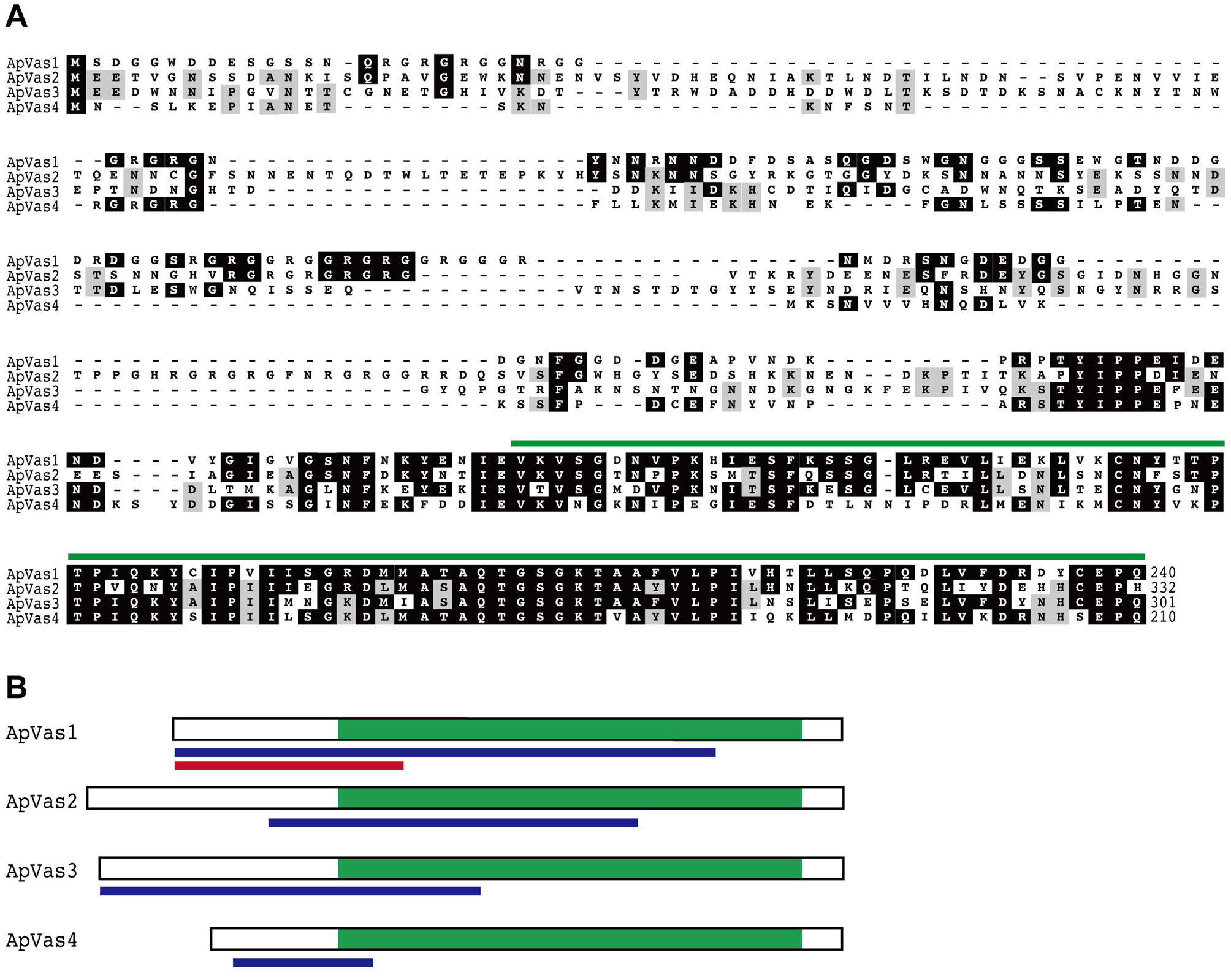

Supplement: Additional file 1: Figure S1 — Sequence alignment of the ApVas1-4 proteins. (A) Alignment of the N-terminal sequences of ApVas1-4. The aligned region includes divergent sequences as well as conserved amino acids in the helicase domain (green bar). Black boxes indicate amino acids identical to that of ApVas1; gray boxes indicate more than two amino acids that are identical between ApVas2-4 proteins. (B) Schematic comparison of ApVas1-4. Green boxes highlight the conserved helicase domain and blue bars mark the locations of the antigen sequences. The red bar beneath ApVas1 highlights the sequence region of the ligand that was used for affinity purifying the ApVas1 antibody. [file 2041-9139-5-18-S1.tiff]

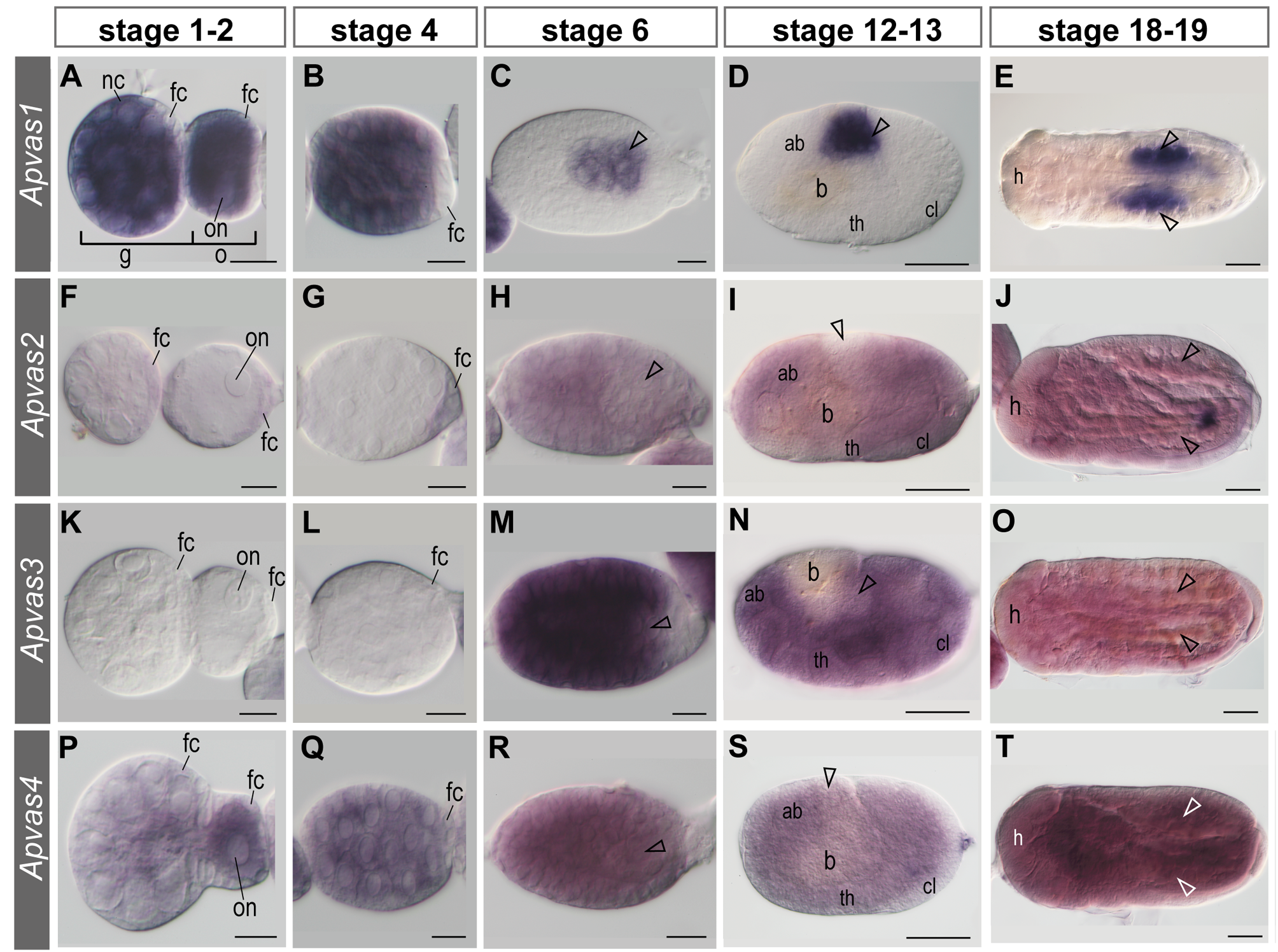

Supplement: Additional file 2: Figure S2 — Expression of Apvas1-4 mRNA during viviparous development. Germaria, oocytes, and embryos in the ovariole were hybridized with the antisense riboprobes of Apvas1-4. Locations of germ cells are indicated with hollow arrowheads. Anterior of egg chambers is to the left. (A-E) Apvas1. (A) In germaria and oocytes, Apvas1 mRNA was expressed in the cytoplasm of nurse cells and oocytes. (B) During nuclear divisions, expression of Apvas1 remained in the cytoplasm of the syncytial blastoderm. (C-E) Expression of Apvas1 was specifically restricted to the germ cells from blastoderm formation (stage 6) till late embryogenesis. (F-J) Apvas2. (F, G) Expression of Apvas2 was undetectable in germaria, oocytes, and syncytial blastoderm, but in the follicle cells weak expression could be identified. (H, I) Except in the germ cells, transcripts of Apvas2 were evenly distributed in embryos before katatrepsis. (J) In embryos after katatrepsis, expression of Apvas2 was ubiquitous including the germ-cell region. (K-O) Apvas3. (K, L) Expression of Apvas3 was not detected in germaria, oocytes, and syncytia (including the follicle cells). (M, N) Like Apvas2 (panels H and I), uniform expression of Apvas3 was detected except in the germ cells. (O) Expression patterns of Apvas3 in late embryos are very similar to those of Apvas2 (panel J). (P-T) Apvas4. (P, Q) Expression of Apvas4 was detected in the germarial lumen, oocytes, and syncytial blastoderm. (R-T) Apvas4 was uniformly distributed in germ cells and somatic cells after blastoderm formation onward. Abbreviations: ab, abdomen; b, bacteria; cl, cephalic lobe; fc, follicle cells; g, germaria; h, head; nc, nurse cells; o, oocytes; on, oocyte nuclei; th, thorax. Scale bars: 20 μm in (A-C, F-H, K-M, P-R), 50 μm in (D, I, N, S) and 100 μm in (E, J, O, T). [file 2041-9139-5-18-S2.tiff]

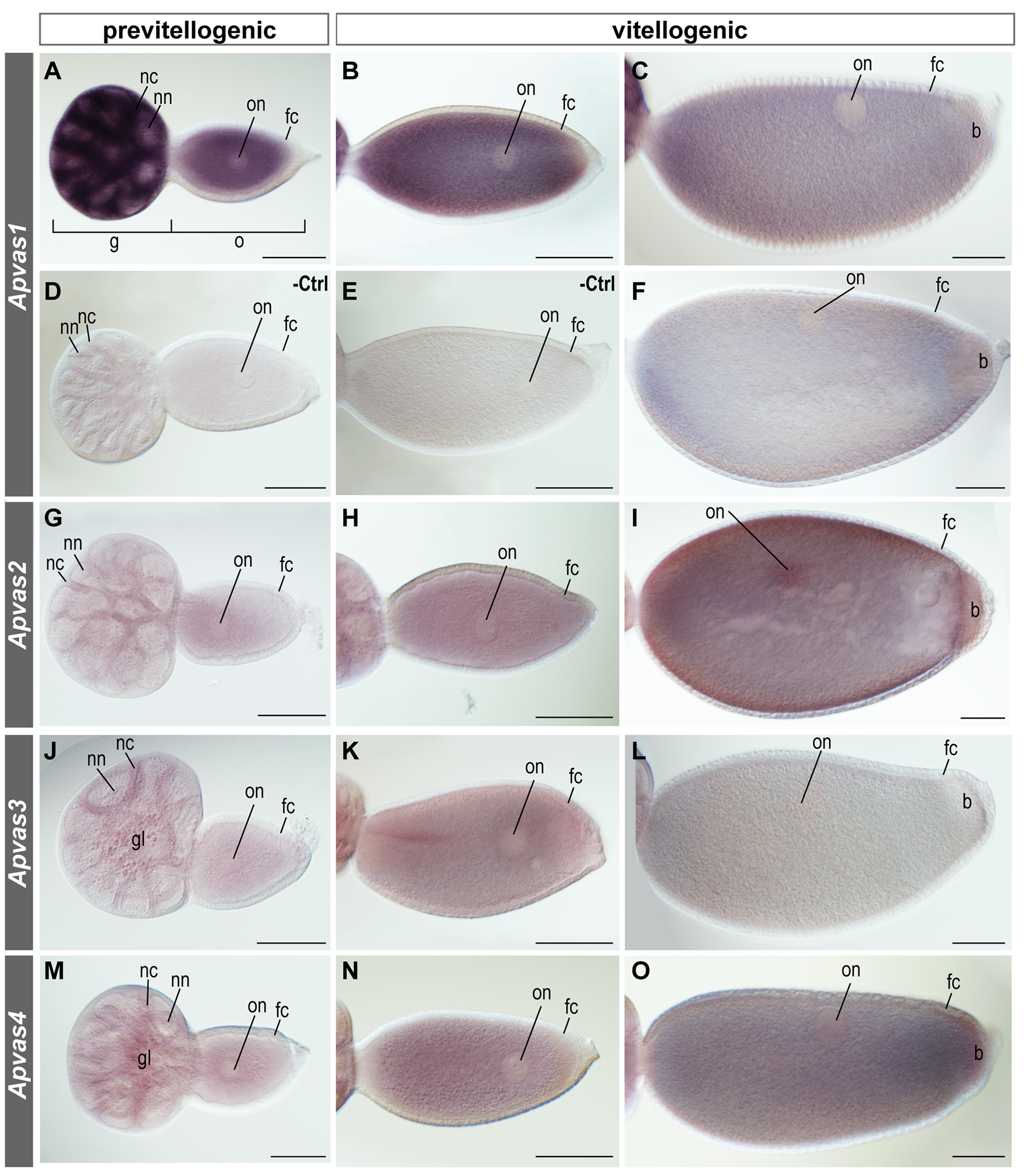

Supplement: Additional file 3: Figure S3 — Expression of Apvas1-4 mRNA in the oviparous ovarioles. (A-F) Apvas1. (A, B, C, F): antisense riboprobes; (D, E): sense riboprobes as negative controls. In germaria and previtellogenic oocytes, Apvas1 mRNA was expressed in the cytoplasm of nurse cells and oocytes (A). During vitellogenesis, expression of Apvas1 remained in the cytoplasm but the intensity of signals decreased as the egg chambers enlarged (B, C, F). (G-I) Apvas2. Expression of Apvas2 was detected in germaria (G), previtellogenic oocytes (G), and early vitellogenic oocytes (H). In late vitellogenic oocytes (I), Apvas2 mRNA was preferentially expressed in the cortex of oocytes as well as the nuclei. (J-L) Apvas3. Expression of Apvas3 was detected in germaria (J), previtellogenic oocytes (J), and early vitellogenic oocytes (K). However, it was almost undetected in late vitellogenic oocytes (L). (M-O) Apvas4. Expression of Apvas4 was detected in germaria (M), previtellogenic oocytes (M), early vitellogenic oocytes (N), and late vitellogenic oocytes (O). Anterior of egg chambers is to the left. Abbreviations: b, bacteria; -Ctrl, negative control; fc, follicle cells; g, germaria; gl, germarial lumen; nc, nurse cells; nn, nurse-cell nuclei; o, oocytes; on, oocyte nuclei. Scale bars: 100 μm. [file 2041-9139-5-18-S3.tiff]

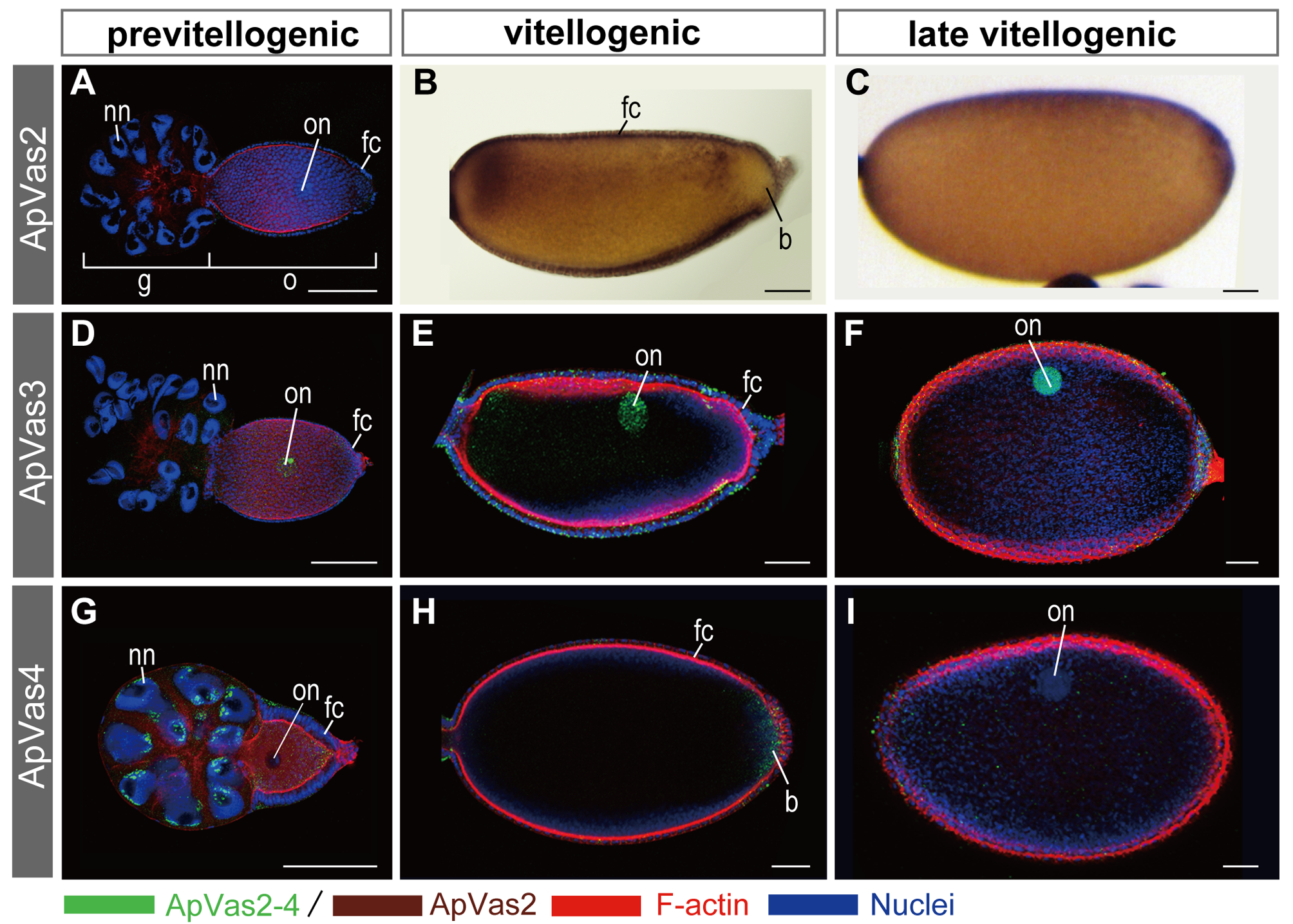

Supplement: Additional file 4: Figure S4 — Expression of ApVas2-4 proteins in the ovarioles dissected from the oviparous females. (A-C) ApVas2. (A) Signals of ApVas2 were not detected in the germaria and developing oocytes. (B, C) During vitellogenesis, expression of ApVas2 was detected in the oocytes and follicle cells. Development of signals was performed using the chromogenic substrate 3,3’-Diaminobenzidine (DAB) Liquid Substrate System (Sigma). (D-F) ApVas3. Expression of ApVas3 was detected in the nuclei throughout oogenesis. (D) Signals of ApVas3 were barely detected in the germaria. (E) ApVas3 expression was restricted to the follicle cells during mid vitellogenesis. (F) In the late vitellogenic oocytes, expression of ApVas3 was almost undetectable. (G-I) ApVas4. (G) Expression of ApVas4 was identified in germaria and previtellogenic oocytes. (H) ApVas4 was preferentially expressed in the oocyte posterior but, unlike ApVas3, ApVas4 expression was not detected in follicle cells and oocyte nuclei. (I) Signals of ApVas4 were ubiquitously distributed within the egg chamber but preferential expression of ApVas4 in the posterior region of oocytes was not detected. Color keys for staining signals of ApVas2-4 (immunostaining), F-actin (Rhodamine Phalloidin), and nuclear DNA (DAPI) are highlighted under the figure. Anterior of egg chambers is to the left. Abbreviations: b, bacteria; fc, follicle cells; g, germaria; nn, nurse-cell nuclei; o, oocytes; on, oocyte nuclei. Scale bars: 100 μm. [file 2041-9139-5-18-S4.tiff]

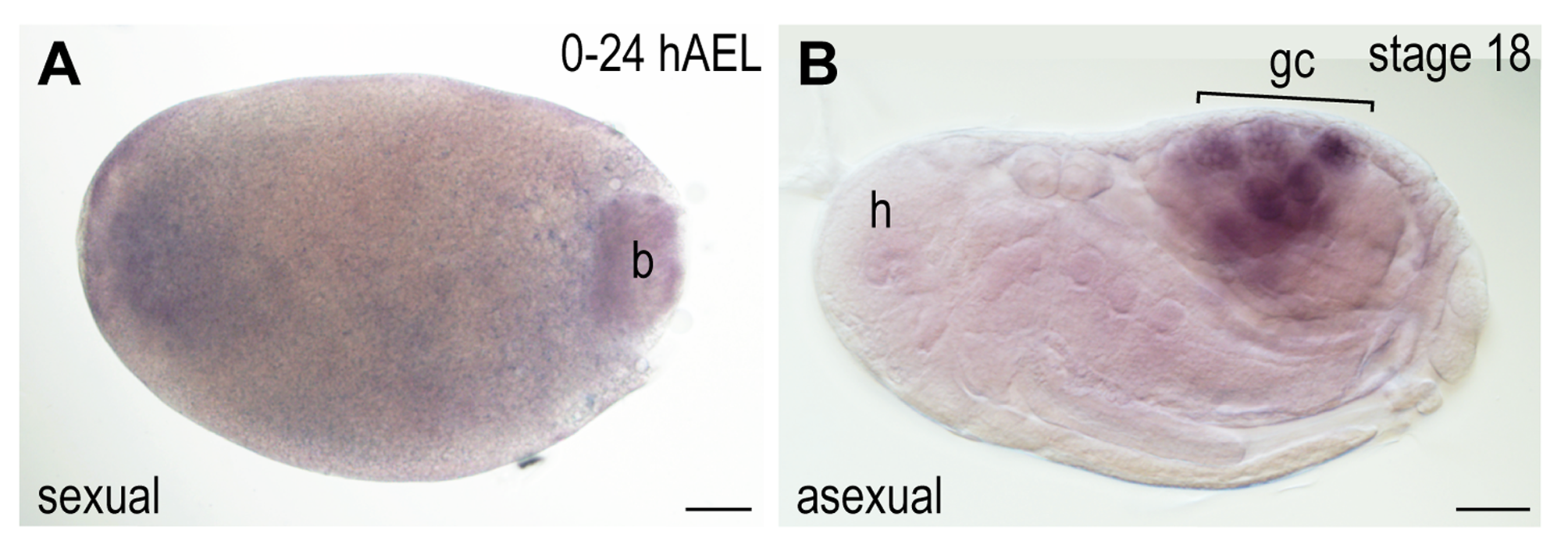

Supplement: Additional file 5: Figure S5 — Expression of Apvas1 mRNA in early oviparous embryos and late viviparous embryos. Embryos were hybridized with antisense riboprobes of Apvas1. (A) Oviparous eggs collected within 24 hAEL. Apvas1 was ubiquitously expressed within the embryos. (B) Viviparous embryos at stage 18 of development. Apvas1 marked germ cells settled in the dorsal region of embryos [42], showing that Apvas1 is germline specific. This staining served as a positive control for probe quality and in situ conditions. Anterior of oviparous egg chambers and viviparous embryos are to the left. Abbreviations: b, bacteria; gc, germ cells; h, head. Scale bars: 100 μm. [file 2041-9139-5-18-S5.tiff]

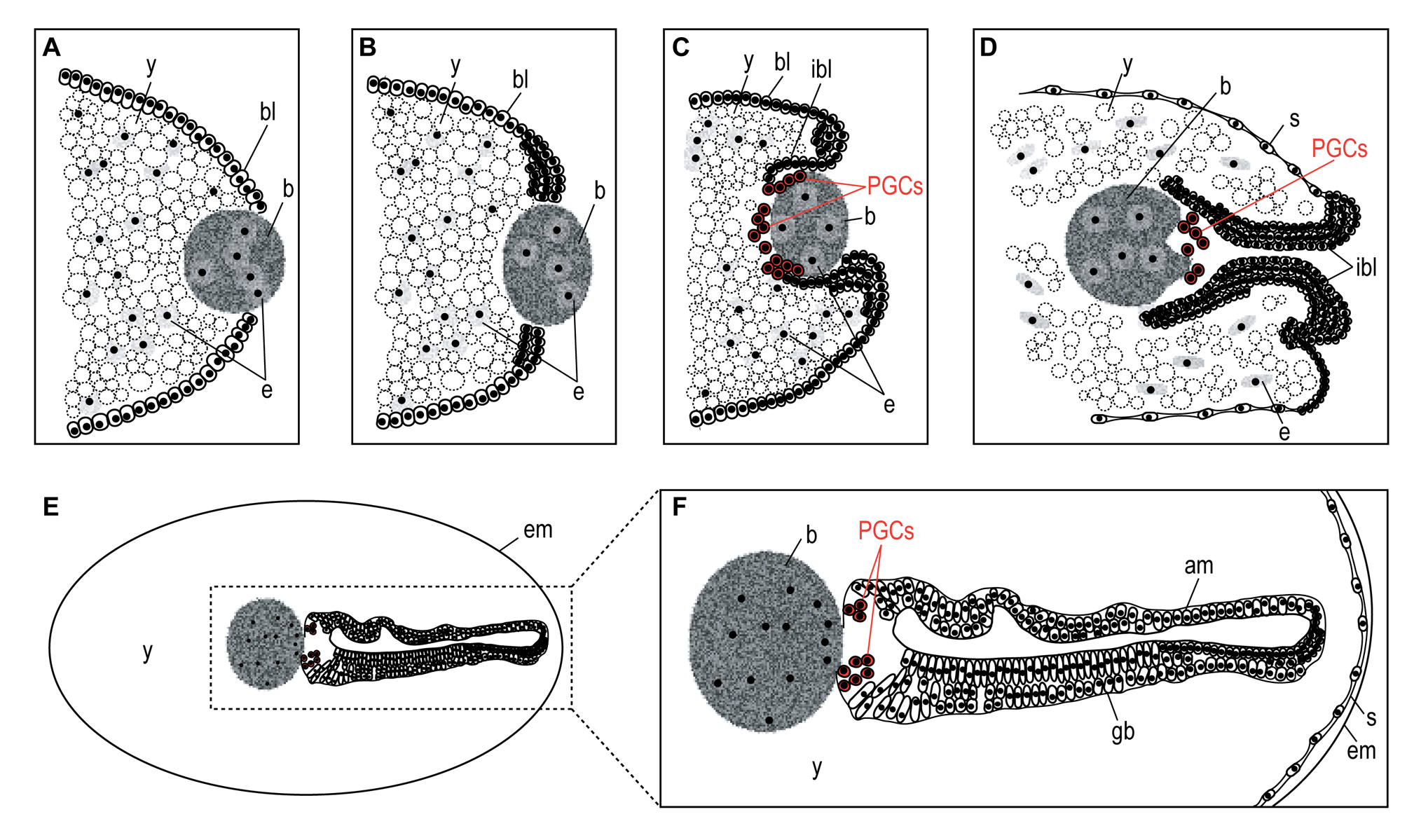

Supplement: Additional file 6: Figure S6 — Schematic illustrations of germ band formation in sexual females. Images in this figure are modifications of Webster and Phillips [16] and Tannreuther [15], where embryogenesis of the spring grain aphid T. graminum and the black willow aphid Melanoxanthus spp. (a synonym of Pterocomma spp.), respectively, are described. (A-C) Invagination of the blastoderm. (A) Before invagination, the single layer of blastodermal cells has been invaded by the endosymbiotic bacteria Buchnera from the posterior pole of the egg. (B) Invagination of the blastodermal cells occurs in the area flanking the bacteria. (C) Invaginating blastoderm and invading bacteria are both migrating into the egg chamber. (D-F) Formation of the germ band. (D) Invaginating blastoderm becomes thickened and extends further inside the yolk. Blastodermal cells that link to the bilateral invaginating blastoderm differentiate into serosal cells. (E, F) Both ends of the extending blastoderm fuse together, detaching from the serosal membrane. One side of the germ band further thickens to become the germ band; another side differentiates into the amnion. Location of ‘primitive germ cells (red)’ corresponds to that of the primordial germ cells expressing ApVas1/Apvas1 (Figure 4M-O; Figure 5A-C). Abbreviation: am, amnion; b, bacteria; bl, blastoderm; e, energids; em, egg membrane; gb, germ band; ibl, invaginating blastoderm; PGCs, primordial germ cells; s, serosa; y, yolk. [file 2041-9139-5-18-S6.tiff]

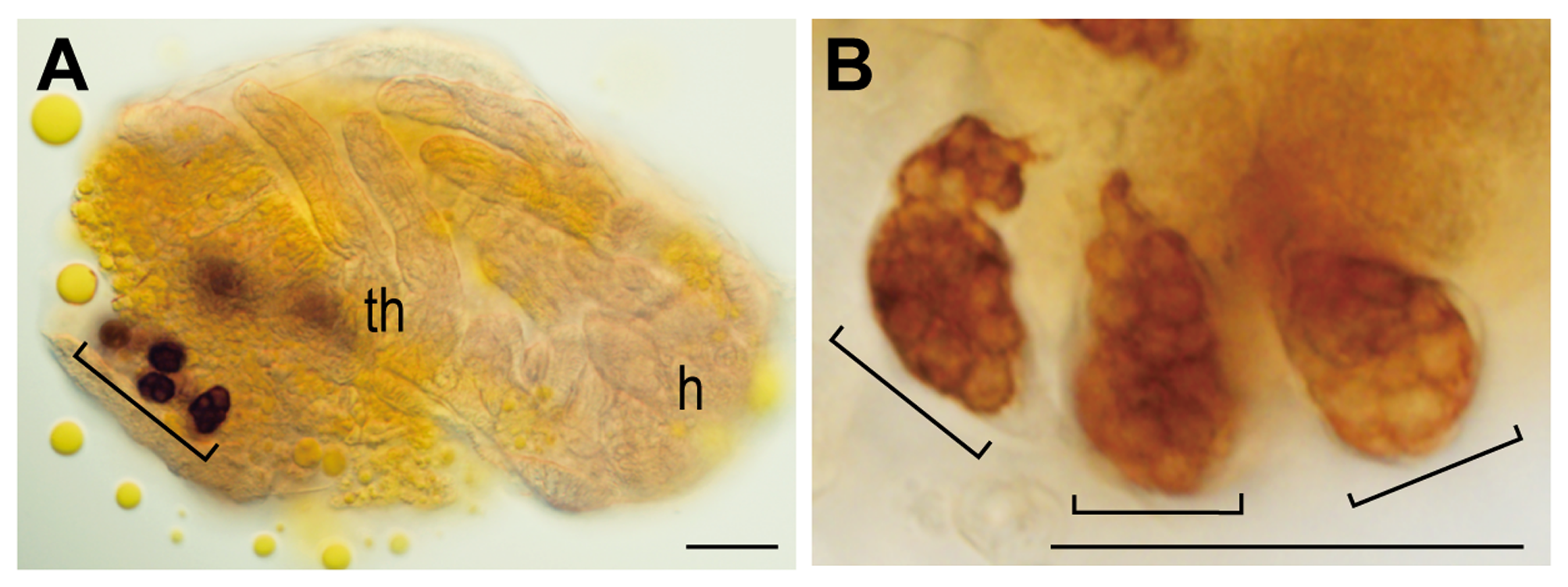

Supplement: Additional file 7: Figure S7 — Expression of ApVas1 in the oviparous embryos undergoing katatrepsis. Anterior of egg chamber is to the left; dorsal side of the embryo is lower. ApVas1-positive cells: brackets. (A) Lateral view of an embryo collected by the end of 35 dAEL. ApVas1-positive cells were bilaterally located in the dorsal region of the abdomen. (B) Magnification of ApVas1-positive cells from another embryo that was also collected by the end of 35 dAEL. As shown in the figure, there are 12 to 13 PGCs within each cluster (brackets). Abbreviations: h, head; th, thorax. Scale bars: 100 μm. [file 2041-9139-5-18-S7.tiff]

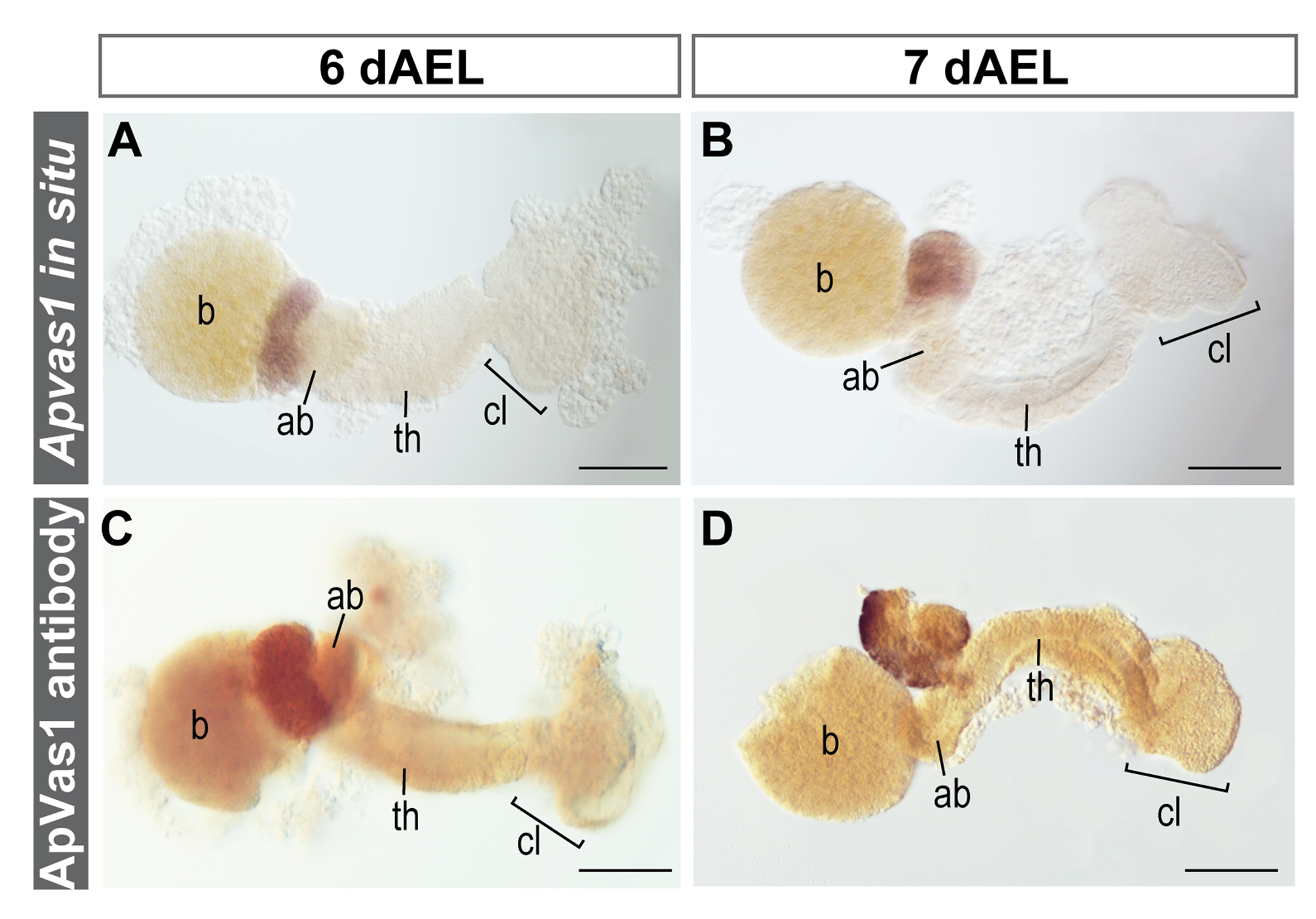

Supplement: Additional file 8: Figure S8 — Expression of Apvas1 mRNA and ApVas1 protein in oviparous eggs collected during 6 to 7 dAEL. From 6 dAEL onward, an enlarged cephalic lobe of the embryo was observed. In the images shown, cephalic lobes of embryos are to the right (though anterior of egg chambers is still to the left). (A, B) Embryos hybridized with antisense riboprobes of Apvas1. (A) Embryos at 6 dAEL. Apvas1-positive cells were aligned within a stripe between bacteria and the embryo (germ band). (B) Embryos at 7 dAEL. Apvas1-positive cells were aggregated as a ball-like shape. (C, D) Embryos were stained with antibody against ApVas1 protein. Expression patterns of ApVas1 (C, D) are very similar to those of Apvas1 (A, B). Abbreviations: ab, abdomen; b, bacteria; cl, cephalic lobe; th, thorax. Scale bars: 100 μm. [file 2041-9139-5-18-S8.tiff]
